# Vac8 spatially confines autophagosome formation at the vacuole in *S. cerevisiae*

**DOI:** 10.1242/jcs.235002

**Published:** 2019-11-14

**Authors:** David M. Hollenstein, Rubén Gómez-Sánchez, Akif Ciftci, Franziska Kriegenburg, Muriel Mari, Raffaela Torggler, Mariya Licheva, Fulvio Reggiori, Claudine Kraft

**Affiliations:** 1 Department of Biochemistry and Cell Biology, Max Perutz Labs, University of Vienna, Vienna BioCenter, Dr. Bohr-Gasse 9, 1030 Vienna, Austria; 2 Institute of Biochemistry and Molecular Biology, ZBMZ, Faculty of Medicine, University of Freiburg, 79104 Freiburg, Germany; 3 Faculty of Biology, University of Freiburg, 79104 Freiburg, Germany; 4 CIBSS - Centre for Integrative Biological Signalling Studies, University of Freiburg; 5 Department of Biomedical Sciences of Cells & Systems, University of Groningen, University Medical Center Groningen, 9713 AV Groningen, The Netherlands

**Keywords:** Autophagy, Vac8, Autophagosome, Phagophore assembly site, PAS

## Abstract

Autophagy is initiated by the formation of a phagophore assembly site (PAS), the precursor of autophagosomes. In mammals, autophagosome formation sites form throughout the cytosol in specialized subdomains of the endoplasmic reticulum (ER). In yeast, the PAS is also generated close to the ER, but always in the vicinity of the vacuole. How the PAS is anchored to the vacuole and the functional significance of this localization are unknown. Here, we investigated the role of the PAS–vacuole connection for bulk autophagy in the yeast *Saccharomyces cerevisiae*. We show that Vac8 constitutes a vacuolar tether that stably anchors the PAS to the vacuole throughout autophagosome biogenesis via the PAS component Atg13. *S. cerevisiae* lacking Vac8 show inefficient autophagosome–vacuole fusion, and form fewer and smaller autophagosomes that often localize away from the vacuole. Thus, the stable PAS–vacuole connection established by Vac8 creates a confined space for autophagosome biogenesis between the ER and the vacuole, and allows spatial coordination of autophagosome formation and autophagosome–vacuole fusion. These findings reveal that the spatial regulation of autophagosome formation at the vacuole is required for efficient bulk autophagy.

## Abstract (precis)

**Highlighted Article:** Vac8 tethers the phagophore assembly site to the vacuole, which creates a confined space for autophagosome biogenesis and coordinates autophagosome formation with vacuolar fusion.

## INTRODUCTION

Macroautophagy, hereafter referred to as autophagy, is an evolutionarily conserved process in eukaryotic cells that enables degradation and recycling of bulk cytoplasmic components. It is the main pathway for the turnover of aggregated proteins, as well as damaged and superfluous organelles, and for providing nutrients during starvation conditions. During autophagy, cargo destined for degradation becomes enwrapped in a double-membrane called the phagophore (also known as the isolation membrane). The phagophore expands around the cargo until its edges close to form a sealed, double-membrane vesicle, the autophagosome. Ultimately, the completed autophagosome fuses with the lytic compartment (the vacuole in yeast and plants, the lysosome in mammals), resulting in degradation of the cargo. In mammalian cells, autophagosomes form in phosphatidylinositol 3-phosphate (PIP3)-rich subdomains of the ER, called omegasomes (Mercer et al., 2018). Similarly, in budding yeast, a connection between the phagophore and the ER is required for autophagosome biogenesis (Gómez-Sánchez et al., 2018; Graef et al., 2013). Moreover, autophagosomes in yeast form in close vicinity to the vacuole (Graef et al., 2013; Suzuki et al., 2013).

Autophagy can operate in a selective and non-selective manner. Selective autophagy involves the specific recognition of a cargo, for example, damaged mitochondria, by dedicated receptors. These receptors subsequently recruit the core autophagy machinery and promote the *in situ* formation and expansion of the growing phagophore around the cargo (Kraft et al., 2009). Conversely, cytoplasmic material is randomly sequestered by autophagosomes during non-selective bulk autophagy. Bulk autophagy is strongly induced upon starvation conditions to provide amino acids and other nutrients required for cellular survival. Therefore, autophagy constitutes a critical mechanism to maintain cellular homeostasis.

The initial step in autophagy is the formation of the phagophore assembly site (PAS, also called the pre-autophagosomal structure), which defines where the phagophore and, ultimately, the autophagosome form. The assembly of the PAS is hierarchical and involves the recruitment of several autophagy-related (Atg) proteins (Suzuki et al., 2007). During selective autophagy in budding yeast, the PAS assembles on the cargo at the vacuole, resulting in local activation of the serine-threonine protein kinase Atg1 (Torggler et al., 2016). In bulk autophagy, however, a specific cargo is not available to serve as a PAS assembly platform. Instead, Atg1 assembles into a pentameric complex with Atg13, Atg17, Atg29 and Atg31. These pentameric complexes further interact with each other resulting in a higher-order oligomeric structure that constitutes the early PAS for bulk autophagy (Yamamoto et al., 2016). Clustering of the Atg1 complex leads to the activation of Atg1 kinase and recruitment of further Atg proteins. Thereby the PAS matures to a site where the phagophore can form. Initially, Atg9 vesicles and the autophagy-specific phosphoinositide 3-kinase (PI3K) complex containing Atg14 are recruited. Subsequently, the Atg2–Atg18 module and the Atg8 lipidation machinery, which consists of the Atg5–Atg12 conjugate and Atg16, are recruited independently (Suzuki et al., 2007). Atg2 appears to be important for establishing the connection between the phagophore and the ER, both during selective and bulk autophagy (Gómez-Sánchez et al., 2018; Kotani et al., 2018). In contrast, however, it remains unclear how the PAS and growing autophagosomes are anchored to the vacuole, and whether this connection fulfills a functional role during autophagosome formation (Suzuki and Ohsumi, 2010).

Vac8 is a vacuolar membrane protein, anchored to lipid bilayers via myristoylation of a glycine residue and palmitoylation of three cysteine residues in its N-terminus (Wang et al., 1998). Vac8 plays a crucial role in vacuole inheritance (Wang et al., 1998), homotypic vacuole fusion (Veit et al., 2001) and establishment of nucleus–vacuole junctions (Pan et al., 2000). Deletion of *VAC8* therefore results in an altered vacuolar morphology, visible as multi-lobed vacuoles.

The crystal structure of Vac8 bound to Nvj1 revealed that Vac8 comprises 12 armadillo repeat domains, organized into a superhelical structure that serves as a protein binding platform (Jeong et al., 2017). Vac8 is known to associate with the Atg1 complex via Atg13 and it has been reported to be involved in bulk autophagy (Scott et al., 2000). However, Vac8 has mainly been associated with selective autophagy, such as the cytoplasm-to-vacuole targeting (Cvt) pathway and piecemeal autophagy of the nucleus (Cheong et al., 2005; Roberts et al., 2002). Despite its characterized roles in vacuolar functions, the function of Vac8 in autophagy is largely unknown.

In this study, we show that Vac8 plays a direct and important role in bulk autophagy. It acts early in the pathway by regulating PAS assembly, as well as during later steps of autophagosome formation and fusion with the vacuole. In the absence of Vac8, autophagosome formation takes place in vicinity to the ER, but a stable vacuolar connection is lost, suggesting that Vac8 is required for tethering the PAS and forming autophagosomes to the vacuole. Moreover, we show that Vac8 tethering of the PAS is mediated by Atg13. Together, our findings show that Vac8 helps to confine and coordinate autophagosome formation between the ER and the vacuole.

## RESULTS

### Vac8 plays a direct and essential role during bulk autophagy

**Fig. 1.**
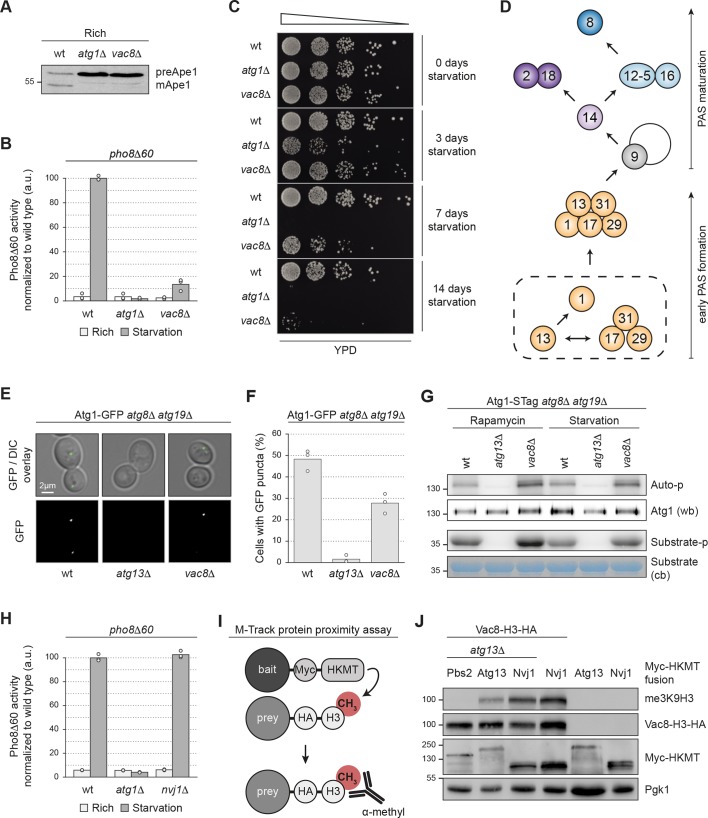
**Vac8 has a role in early bulk PAS formation.** (A) The indicated strains were grown to mid-log phase in YPD medium and cell extracts were prepared by TCA precipitation. Ape1 processing was analysed by anti-Ape1 western blotting. One representative experiment out of three is shown. (B) The indicated strains were grown to mid-log phase in YPD medium and subsequently starved for 4 h in SD-N medium where indicated. Pho8Δ60 alkaline phosphatase activity was measured in three independent experiments. The values of each replicate (circles) and the mean (bars) were plotted. All values were normalized to the mean Pho8Δ60 alkaline phosphatase activity of the wild type. (C) Wild-type, *atg1*Δ *and vac8*Δ cells were grown to mid-log phase in YPD medium and shifted to SD-N medium to induce starvation. After 0, 3, 7 and 14 days, cells were spotted in serial dilutions onto YPD plates and incubated for 48 h at 30°C. One representative experiment out of four is shown. (D) Model of hierarchical recruitment of bulk PAS factors, originally described by Suzuki et al. (2007). (E,F) Strains containing endogenously expressed Atg1–GFP as indicated were grown to mid-log phase in SD medium and starved for 1 h in SD-N medium. The number of cells with Atg1–GFP puncta was counted in three independent experiments. For each strain and replicate, at least 100 cells were analysed. (E) Representative microscopy images. (F) Quantification of the percentage of cells with at least one Atg1–GFP punctum. The values of each individual experiment (circles) and the mean (bars) were plotted. DIC, differential interference contrast. (G) The indicated strains were grown to mid-log phase in YPD medium and treated for 1 h with 220 nM rapamycin or starved for 1 h in SD-N medium. Endogenously expressed Atg1-STag (Atg1-S-peptide-2×ProteinA) was immunoprecipitated and incubated with γ-[^32^P]ATP and substrate (Atg19 C-terminus; Pfaffenwimmer et al., 2014). Kinase activity was measured through autoradiography detecting Atg1 auto-phosphorylation and substrate phosphorylation. Atg1–STag levels were assessed by anti-ProteinA (PAP) western blotting (wb) and substrate levels were analysed through Coomassie Blue staining (cb). One representative experiment out of three is shown. (H) Indicated strains were grown to mid-log phase in YPD medium and subsequently starved for 4 h in SD-N medium where indicated. Pho8Δ60 alkaline phosphatase activity was measured in three independent experiments. The values of each replicate (circles) and the mean (bars) were plotted. All values were normalized to the mean Pho8Δ60 alkaline phosphatase activity of the wild type. (I) Schematic illustration of the M-track protein proximity assay. The bait protein is tagged with a histone lysine methyltransferase (HKMT) and the prey protein with a histone 3 (H3) domain. Upon close proximity of bait and prey, the H3 domain becomes stably methylated by the HKMT, which can be detected by using an antibody specific for methylated H3. (J) Indicated strains carrying a plasmid expressing Myc–HKMT-tagged Atg13, Nvj1 or, as a control, Pbs2, and either endogenously expressed H3–HA-tagged Vac8 or untagged Vac8 were grown to mid-log phase in SD medium and treated for 4 h with 220 nM rapamycin. Cell extracts were prepared by TCA precipitation. The Vac8–H3–HA tri-methylation signal was assessed by anti-me3K9H3 western blotting. Vac8–H3–HA expression levels, Myc–HKMT-tagged Atg13, Pbs2 or Nvj1 expression levels and loading were monitored by anti-HA, anti-Myc and anti-Pgk1 western blotting, respectively. One representative experiment out of three is shown. a.u., arbitrary units; wt, wild type.

Previous reports have described Vac8 as essential for the selective Cvt pathway, but less important for bulk autophagy, although conflicting conclusions exist (Scott et al., 2000). Its mechanistic role in autophagy, however, remains unknown. To address this question, we first revisited the involvement of Vac8 in the Cvt pathway. As expected, Ape1 processing via the Cvt pathway was strongly impaired in *vac8*Δ and in *atg1*Δ mutant cells compared to that in wild-type cells (Scott et al., 2000; Fig. 1A). To assess bulk autophagy, we monitored the activity of Pho8Δ60 reporter phosphatase, which requires bulk autophagy for delivery to and enzymatic activation in the vacuole (Noda et al., 1995). Pho8Δ60 activity was substantially lower in both *vac8*Δ and *atg1*Δ mutant cells compared to wild-type cells, as previously shown (Fig. 1B) (Cheong et al., 2005; Scott et al., 2000). Bulk autophagy is required for cell survival during starvation (Tsukada and Ohsumi, 1993), *vac8*Δ mutants should therefore be starvation sensitive. Wild-type, *atg1*Δ and *vac8*Δ cells were nitrogen-starved for several days followed by serial dilution spotting onto nutrient rich agar plates to test cell viability. Whereas wild-type cells were able to cope with nitrogen starvation, *atg1*Δ control cells displayed a severe starvation sensitivity (Fig. 1C). *vac8*Δ cells were also starvation sensitive, especially after prolonged starvation, which is in line with the severe bulk autophagy defect of these mutants.

Vacuolar integrity is important for late steps of autophagy, such as the fusion of autophagosomes with the vacuole and the breakdown of the autophagic cargo in the vacuolar lumen. Therefore, a defect in vacuolar function or integrity could indirectly affect autophagy. Indeed, *vac8*Δ cells show a severe defect in vacuole–vacuole fusion and contain fragmented vacuoles (Pan and Goldfarb, 1998; Veit et al., 2001).

We reasoned that if the bulk autophagy defect in *vac8*Δ mutants arises from abnormal vacuolar morphology only, then early steps of autophagy should be unaffected. To test this hypothesis, we determined whether the initiation of autophagy was affected in *vac8*Δ mutants. Specifically, we used a fluorescent Atg1–GFP reporter to detect the formation of the Atg1 complex within the early PAS (Fig. 1D) (Suzuki et al., 2007). Importantly, to monitor bulk autophagy only, we used cells lacking the receptor Atg19 or the cargo Ape1, which thus prevents Cvt pathway-induced PAS formation. Furthermore, we used an *atg8*Δ background, which means cells are unable to form autophagic membranes; this results in stalling the PAS and prevents its turnover. This experimental setup allows the specific analysis of early PAS formation during bulk autophagy.

We observed Atg1–GFP puncta in 50% of *atg8*Δ*atg19*Δ control cells after 1 h of starvation, but in less than 2% of negative control cells that lack the Atg1 complex component Atg13 (*atg13*Δ*atg8*Δ*atg19*Δ) (Fig. 1E,F). Importantly, only 25% of *vac8*Δ*atg8*Δ*atg19*Δ cells displayed Atg1–GFP puncta, suggesting that Vac8 is required for an early step of autophagy, namely during PAS formation.

Because Vac8 associates with the Atg1 complex via Atg13, we considered that Vac8 influences Atg1 kinase activation during bulk autophagy. To test this possibility, we immunopurified Atg1–STag and performed an *in vitro* kinase assay. As expected, Atg13 was required for Atg1 kinase activation. However, the deletion of Vac8 did not diminish Atg1 kinase activity during bulk autophagy-inducing conditions, suggesting that the bulk autophagy defect in *vac8*Δ mutants is caused by other means than reduced Atg1 kinase activity (Fig. 1G).

Another interaction partner of Vac8 is Nvj1. Binding of Nvj1 to Vac8 is required for establishing nucleus–vacuole junctions (NVJ), which generates a connection between the vacuole and nuclear ER (Pan et al., 2000). To test whether the defect in PAS formation of *vac8*Δ mutants is caused by impaired NVJ formation, we tested whether *nvj1*Δ mutants are defective in bulk autophagy. In a Pho8Δ60 assay, *nvj1*Δ cells showed no defect in bulk autophagy (Fig. 1H). Similarly, when monitoring the cleavage of Pgk1–GFP, an assay that also is used to monitor bulk autophagy flux (Welter et al., 2010), both wild-type and *nvj1*Δ cells showed similar levels of cleaved GFP after 4 h of nitrogen starvation (Fig. S1), suggesting that Nvj1 is dispensable for bulk autophagy function and therefore does not influence PAS formation.

*In vitro* analysis suggests that Nvj1 and Atg13 bind to the same region on Vac8 in an exclusive and competitive manner (Jeong et al., 2017), suggesting that Nvj1 and Atg13 are unlikely to assemble into the same holo complex with Vac8. To test whether Nvj1 also interacts with Vac8 in the absence of Atg13 *in vivo*, we evaluated this interaction using the methylation tracking (M-track) proximity assay. The approach employs a histone lysine methyltransferase (HKMT) domain fused to a bait protein, and the methylation acceptor histone 3 (H3) fused to a prey protein. Upon binding to the bait, the prey is stably methylated *in vivo*, and this modification is detected using anti-methylation-specific antibodies (Fig. 1I) (Brezovich et al., 2015; Zuzuarregui et al., 2012). We fused Atg13 and Nvj1 to the HKMT domain, and Vac8 to the H3 tag. As expected, we observed methylation of Vac8–H3 with Atg13–HKMT. In addition, Nvj1–HKMT interacted with Vac8–H3 in *atg13*Δ cells, confirming that Nvj1 interacts with Vac8 independently of Atg13 *in vivo* (Fig. 1J).

Together, these results suggest that Vac8, independently of its function in NVJ formation, plays a direct and important role in bulk autophagy that involves the formation of the early PAS.

### Vac8 regulates early PAS formation but not PAS maturation

Although *vac8*Δ mutants showed a 50% reduction in early PAS formation, the Pho8Δ60 assay suggests that bulk autophagic flux is almost completely impaired in *vac8*Δ cells (Fig. 1B). This apparent discrepancy suggests that Vac8 might also regulate later steps in autophagy, after establishing the early PAS.

**Fig. 2.**
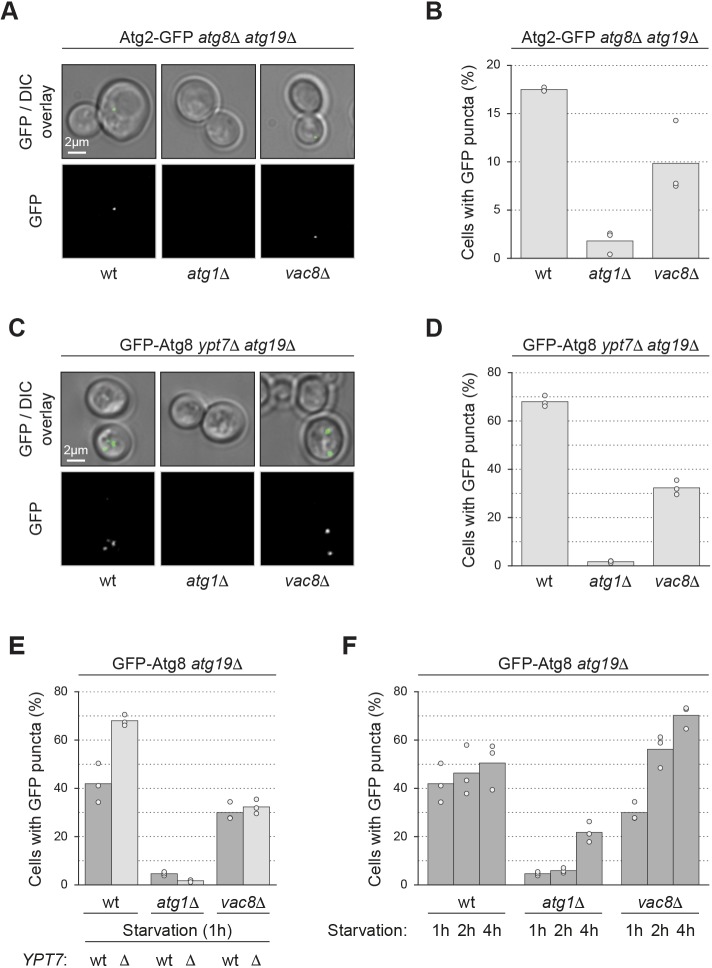
**Vac8 has an additional function post bulk PAS assembly.** (A,B) The indicated strains containing endogenously expressed Atg2–GFP were grown to mid-log phase in SD medium and starved for 1 h in SD-N medium. The number of cells with Atg2–GFP puncta was counted in three independent experiments. For each strain and replicate, at least 100 cells were analysed. (A) Representative microscopy images. (B) Quantification of the percentage of cells with at least one Atg2–GFP punctum. The values of each replicate (circles) and the mean (bars) were plotted. (C,D) Analysis of fluorescent GFP–Atg8 punctum formation in *vac8*Δ cells after starvation. GFP–Atg8 was expressed endogenously. (C) Representative microscopy images and (D) quantification. Experiments were performed and presented as described in B. (E,F) Analysis of GFP–Atg8 punctum turnover in *vac8*Δ cells during starvation. Experiments were performed and presented as described in B; strains were starved for 1, 2 or 4 h in SD-N medium. Data from *ypt7*Δ mutants (light grey bars) shown in E correspond to the experiments shown in D; data from *YPT7* wild type (dark grey bars) shown in E correspond to the experiments shown in F. DIC, differential interference contrast; wt, wild type.

The early PAS formed by the Atg1 complex subsequently matures via the recruitment of downstream Atg proteins. First, Atg9 and Atg14 join the PAS, followed by the independent recruitment of the Atg2–Atg18 module as well as the Atg12 and Atg8 conjugation systems (Suzuki et al., 2007). We assessed the recruitment of Atg2 to the PAS by monitoring Atg2–GFP localization in *atg8*Δ*atg19*Δ cells. The frequency of cells with Atg2–GFP puncta was reduced by 50% in *vac8*Δ mutants compared to Vac8-containing cells (Fig. 2A,B), similar to the observations with Atg1–GFP.

Additionally, to assess the recruitment of Atg8 to the PAS, we monitored GFP–Atg8 in cells lacking Ypt7 and Atg19. *ypt7*Δ cells are deficient in autophagosome–vacuole fusion and thus accumulate mature autophagosomes (Kirisako et al., 1999). As a result, GFP–Atg8 puncta represent either the PAS or forming autophagosomes. In both cases, GFP–Atg8 puncta indicate that a PAS had been initiated. Similar to what is seen for Atg1–GFP and Atg2–GFP, the frequency of cells with GFP–Atg8 puncta was reduced by 50% in the *vac8*Δ mutant compared to the corresponding wild type (Fig. 2C,D).

We infer that the reduced recruitment of Atg2–GFP and GFP–Atg8 to the PAS reflects the defect in early PAS formation in the *vac8*Δ mutant. Vac8 therefore regulates early PAS formation, but does not further affect PAS maturation, that is recruitment of downstream Atg proteins to the PAS.

### Vac8 regulates autophagosome turnover

Next, we asked whether autophagosome turnover is affected by Vac8. To evaluate autophagosome turnover, we compared the frequency of cells with GFP–Atg8 puncta in Ypt7-containing cells versus fusion-deficient *ypt7*Δ mutants after 1 h of starvation. As expected, the frequency of cells with GFP–Atg8 puncta increased from 40% in Ypt7-containing *atg19*Δ cells to almost 70% in fusion-defective *atg19*Δ*ypt7*Δ cells, indicating that in Ypt7-containing cells autophagosomes are turned over by fusion with the vacuole after 1 h of starvation. In contrast, *vac8*Δ*atg19*Δ and *vac8*Δ*atg19*Δ*ypt7*Δ cells displayed a similar frequency of GFP–Atg8 punctum-containing cells after 1 h of starvation (Fig. 2E), suggesting that *vac8*Δ cells are deficient for autophagosome turnover.

To confirm the defect of *vac8*Δ cells in autophagosome turnover, we monitored whether *atg19*Δ and *vac8*Δ*atg19*Δ cells accumulate GFP–Atg8 puncta over a prolonged period of starvation. In fact, the number of cells containing GFP–Atg8 puncta remained almost constant in *atg19*Δ cells, whereas a substantial increase in the *vac8*Δ*atg19*Δ mutant was observed during 4 h of starvation (Fig. 2F). This supports the hypothesis that *vac8*Δ cells are inefficient in turning over GFP–Atg8 structures. This phenotype could be caused by a kinetic defect in phagophore formation and autophagosome completion, or be due to inefficient fusion of autophagosomes with the vacuole.

Together, these findings demonstrate that in addition to early PAS formation, Vac8 also regulates a later step in autophagy, the formation or fusion of autophagosomes.

### Vac8 mutants display smaller autophagosomes and reduced autophagosome-vacuole fusion

**Fig. 3.**
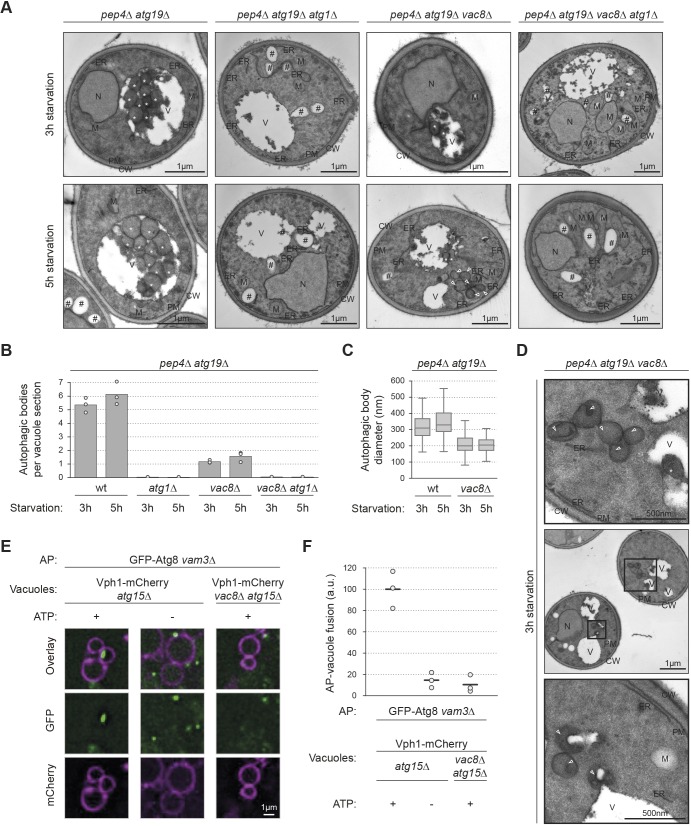
**Deletion of Vac8 results in the delivery of fewer and smaller autophagosomes to the vacuole.** (A–D) The indicated strains were grown to mid-log phase, starved for 3 or 5 h in SD-N medium, fixed in potassium permanganate and analysed by transmission electron microscopy. (A,D) Representative electron micrographs. CW, cell wall; ER, endoplasmic reticulum; M, mitochondria; N, nucleus; PM, plasma membrane; V, vacuole; asterisks, autophagic bodies; #, lipid droplets; arrowheads, autophagosomes. In the *pep4*Δ*atg19*Δ*vac8*Δ mutant, after 3 h of starvation, 85% of cytoplasmic autophagosomes were not connected with the vacuole, whereas 15% of autophagosomes were adjacent to vacuoles (60 cell profiles containing autophagosomes were quantified, the s.e.m. was 1.92). (B) The number of autophagic bodies per vacuole section was quantified in three independent technical replicates. For each condition and replicate 50 cell sections were analysed. The average of each replicate (circles) and the overall mean (bars) were plotted. (C) Boxplot of autophagic body size. At least 90 autophagic bodies from three independent technical replicates were analysed per strain and condition. Dark horizontal lines represent medians, boxes represent the 25th and 75th percentiles, whiskers expand to the largest value no further than 1.5 times the interquartile range; outliers are not shown. (E) Vacuoles were isolated from Vph1–mCherry *atg15*Δ or Vph1–mCherry *atg15*Δ*vac8*Δ cells and incubated with autophagosomal (AP) fractions prepared from GFP–Atg8 *vam3*Δ cells and an energy regeneration system for 2 h. Apyrase was added to deplete ATP in the negative control. Fusion was monitored by fluorescence microscopy and judged by the appearance of a mobile green dot in the vacuole. Shown is the first time point of a 20 s time lapse (see also Fig. S2). (F) Quantification of successful fusion events in (E). The number of successful fusion events per vacuole was counted in three independent experiments and normalized to the positive control, values of each individual experiment (circles) and the mean (horizontal lines) were plotted. a.u., arbitrary units; wt, wild type.

To further assess the role of Vac8 in later steps of autophagy, we analysed *vac8*Δ mutants by transmission electron microscopy. We visualized the accumulation of autophagic bodies in the vacuolar lumen of vacuolar protease mutant *pep4*Δ cells (Guimaraes et al., 2015). As expected, autophagic bodies accumulated in the vacuole of *pep4*Δ*atg19*Δ cells. However, autophagic bodies were not observed in *pep4*Δ*atg19*Δ*atg1*Δ cells, owing to their inability to form autophagosomes (Cheong et al., 2007). The *pep4*Δ*atg19*Δ*vac8*Δ mutant accumulated very few autophagic bodies in the vacuole compared to *pep4*Δ*atg19*Δ cells (Fig. 3A,B), consistent with a highly reduced bulk autophagic flux in the absence of Vac8 (Fig. 1B). Furthermore, autophagic bodies in *pep4*Δ*atg19*Δ*vac8*Δ cells were only 200 nm in diameter, whereas the average diameter of autophagic bodies in *VAC8* wild-type cells was 300 nm, suggesting that deletion of Vac8 impairs the formation of fully sized autophagosomes (Fig. 3A,C).

Unexpectedly, *pep4*Δ*atg19*Δ*vac8*Δ cells accumulated large vesicles in the cytosol (Fig. 3D, arrowheads). These cytosolic structures were not observed in *pep4*Δ*atg19*Δ or in *pep4*Δ*atg19*Δ*atg1*Δ cells. Furthermore, they were not present in *pep4*Δ*atg19*Δ*vac8*Δ*atg1*Δ cells (Fig. 3A), indicating that they correspond to smaller autophagosomes formed in *vac8*Δ cells. These findings are consistent with the observed accumulation of GFP–Atg8 puncta in *vac8*Δ cells after prolonged starvation (Fig. 2F). The autophagosomes that formed in the absence of Vac8 were mostly not connected to the vacuole (85%, Fig. 3A,D), suggesting that Vac8 is involved in targeting or tethering autophagosomes to the vacuolar membrane. Eventually, however, some vacuolar contact must happen, as some fusion also occurred in *vac8*Δ cells.

Next, we asked whether autophagosome–vacuole fusion is affected by Vac8. To test for autophagosome–vacuole fusion, we took advantage of our recently established *in vitro* fusion assay (Bas et al., 2018), which recapitulates autophagosome–vacuole fusion *in vitro* using isolated yeast vacuoles and an autophagosome-enriched fraction. As previously described, autophagosomes were isolated from starved and fusion-deficient GFP–Atg8 *vam3*Δ cells. Vacuoles were isolated from cells lacking the vacuolar lipase Atg15, which results in the stabilization of autophagic bodies within the vacuole and allows their visualization. The vacuolar membrane protein Vph1 was tagged with mCherry to visualize the vacuole. Vacuoles isolated from Vph1–mCherry *atg15*Δ cells were proficient in autophagosome–vacuole fusion, as expected. In the absence of ATP, fusion was inhibited, confirming previous findings that the fusion process requires ATP (Bas et al., 2018). Intriguingly, when vacuoles were isolated from Vph1–mCherry *vac8*Δ*atg15*Δ cells, fusion was blocked, suggesting that Vac8 supports autophagosome–vacuole fusion (Fig. 3E,F; Fig. S2).

### Vac8 membrane association is required for bulk autophagy

**Fig. 4.**
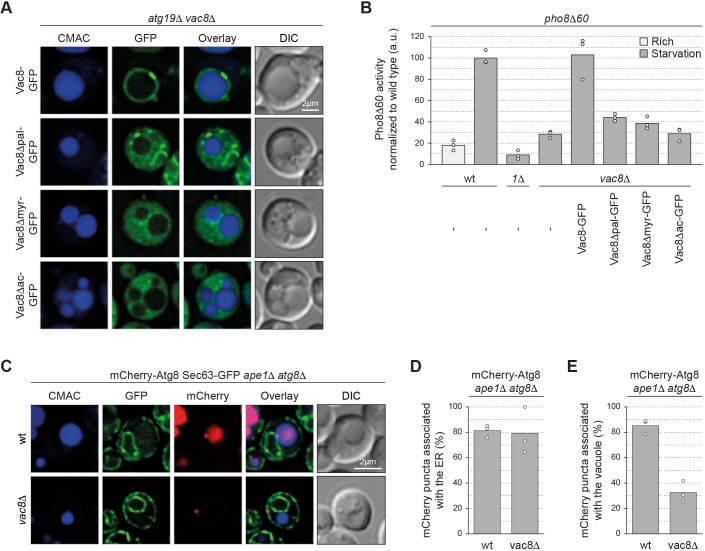
**Vac8 anchors the bulk PAS and forming autophagosomes to the vacuole.** (A) *atg19*Δ*vac8*Δ cells carrying a plasmid expressing Vac8–GFP, Vac8Δpal–GFP (Vac8-C4G-C5T-C7S-GFP), Vac8Δmyr–GFP (Vac8-G2A-GFP) or Vac8Δac–GFP (Vac8-G2A-C4G-C5T-C7S-GFP) were grown to mid-log phase in SD medium, starved for 1 h in SD-N medium and labelled with CMAC. Representative fluorescence images are shown. (B) The indicated strains transformed with a plasmid expressing Vac8–GFP, Vac8Δpal–GFP, Vac8Δmyr–GFP or Vac8Δac–GFP or an empty vector were grown to mid-log phase in SD medium and starved for 4 h in SD-N medium where indicated. Pho8Δ60 alkaline phosphatase activity was measured in three independent experiments. The values of each replicate (circles) and the mean (bars) were plotted. All values were normalized to the mean Pho8Δ60 alkaline phosphatase activity of the wild type. (C–E) Cells containing endogenously expressed Sec63–GFP and plasmid expressed mCherry-V5-Atg8 were grown to mid-log phase in SD medium, starved for 1 h in SD-N medium, and labelled with CMAC. Three independent experiments were performed. For each strain and replicate at least 100 cells were analysed. (C) Representative fluorescence images. The percentage of mCherry–Atg8 puncta associated with (D) the ER and (E) the vacuole was quantified; the values of each replicate (circles) and the mean (bars) were plotted. DIC, differential interference contrast; a.u., arbitrary units; wt, wild type.

Vacuolar membrane anchoring of Vac8 depends on both palmitoylation and myristoylation in its N-terminus (Wang et al., 1998). To determine whether the vacuolar membrane association of Vac8 is important for bulk autophagy, we assessed whether vacuolar localization-defective Vac8 mutants that disrupt the Vac8 myristoylation site (Vac8Δmyr–GFP), the palmitoylation sites (Vac8Δpal–GFP) or all acylation sites (Vac8Δac–GFP) (Wang et al., 1998) could restore Pho8Δ60 activity in *vac8*Δ cells. As expected, all three mutant proteins localized primarily to the cytoplasm in *atg19*Δ*vac8*Δ cells, whereas Vac8–GFP showed vacuolar localization (Fig. 4A). Unlike Vac8–GFP, none of the mutants could substantially restore Pho8Δ60 activity to *vac8*Δ cells (Fig. 4B), suggesting that both myristoylation and palmitoylation are required for bulk autophagy. In contrast to previous observations (Wang et al., 1998), only Vac8–GFP expression fully rescued the Cvt pathway defect in the *vac8*Δ knockout, while *vac8*Δ cells expressing Vac8Δpal–GFP and Vac8Δmyr–GFP displayed mild Cvt pathway defects, and those expressing Vac8Δac–GFP showed a severe Cvt pathway defect (Fig. S3A). These data suggest that vacuolar localization of Vac8 is required for bulk autophagy.

### Vac8 stably anchors the PAS and forming autophagosomes at the vacuole

It has been reported that autophagosomes are simultaneously tethered to the vacuole and the ER during their formation (Graef et al., 2013; Suzuki et al., 2013). Atg2 was recently shown to play a role in connecting the autophagosomes to the ER (Gómez-Sánchez et al., 2018), but the factor(s) that connect the autophagosomes to the vacuole are unknown. Because we observed autophagosomes in the cytosol of the *vac8*Δ mutant (Fig. 3D), we hypothesized that Vac8 could mediate the autophagosome–vacuole connection. To test this possibility, we used mCherry–Atg8 to monitor the localization of the PAS and forming autophagosomes, and quantified their proximity to the vacuole (CMAC) and the ER (Sec63–GFP). mCherry–Atg8-positive structures, which represent both PAS and forming autophagosomes, were associated with the ER in ∼80% of both wild-type and *vac8*Δ cells (Fig. 4C,D). In addition, mCherry–Atg8-positive structures also localized adjacent to the vacuole in ∼80% of wild-type cells, suggesting that PAS assembly and autophagosome formation takes place in between the ER and the vacuole in 60–80% of cells (Fig. 4E), as previously suggested (Graef et al., 2013). However, the vacuolar proximity of mCherry–Atg8 puncta was substantially reduced to ∼30% in *vac8*Δ mutants (Fig. 4E). Similarly, mCherry–Atg8 displayed reduced vacuolar localization in the *vac8*Δ cells expressing cytoplasmic Vac8Δac–GFP (Fig. S3B,C). These findings suggest that Vac8 is required to stably anchor the PAS and/or autophagosomes at the vacuole.

### Ectopic Vac8 recruits the PAS protein Atg13

**Fig. 5.**
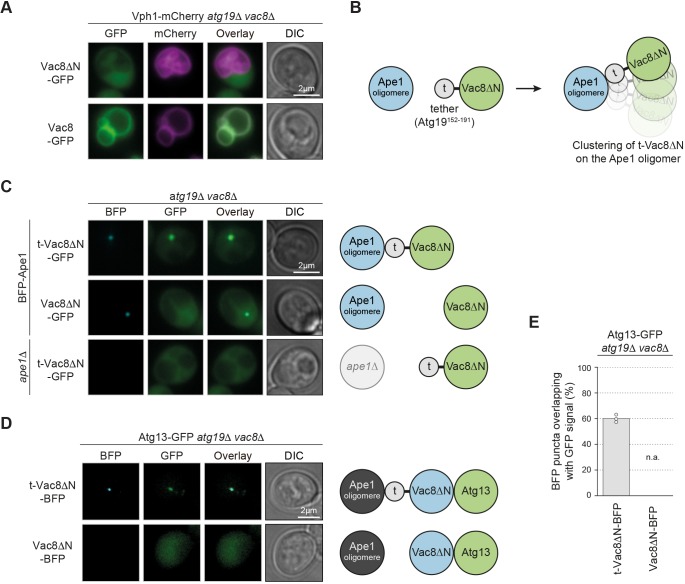
**Vac8 binds to Atg13 *in vivo*.** (A) Vph1–mCherry *atg19*Δ*vac8*Δ cells carrying a plasmid expressing Vac8ΔN–GFP (Vac8^19-578^-GFP) or Vac8–GFP were grown to mid-log phase in SD medium and starved for 1 h in SD-N medium. Representative fluorescence images are shown. (B) Schematic of the experimental setup used in (C). N-terminal truncation of 18 amino acids results in cytosolic distribution of Vac8ΔN (Vac8^19-578^). Fusion of the Ape1-binding region of Atg19^152-191^ to Vac8ΔN (t-Vac8ΔN) results in ectopic localization and clustering of t-Vac8ΔN on Ape1 oligomers. (C) *atg19*Δ*vac8*Δ cells carrying a plasmid expressing BFP–Ape1 and either t-Vac8ΔN–GFP or Vac8ΔN–GFP, and *atg19*Δ*vac8*Δ*ape1*Δ cells carrying an empty plasmid and a plasmid expressing t-Vac8ΔN–GFP were grown to mid-log phase in SD medium and starved for 1 h in SD-N medium. Representative fluorescence images are shown. (D) Atg13–GFP *atg19*Δ*vac8*Δ cells carrying a plasmid expressing t-Vac8ΔN–BFP or Vac8ΔN–BFP were grown to mid-log phase in SD medium and starved for 1 h in SD-N medium. Representative fluorescence images are shown. (E) Quantification of D. The percentage of BFP puncta overlapping with GFP signal was quantified in three independent experiments; the values of each replicate (circles) and the mean (bars) were plotted. t, tether; DIC, differential interference contrast; n.a., not applicable (none visible).

We hypothesized that if endogenous Vac8 tethers the PAS to the vacuole, then ectopic localization of Vac8 should tether the PAS to another site in the cell. To test this hypothesis, we used Ape1 oligomers for ectopic recruitment of Vac8. Ape1 oligomers provide a protein-clustering platform that enables the visualization of recruited proteins by fluorescence microscopy. Importantly, in the absence of the cargo receptor Atg19, the Ape1 oligomer remains cytosolic and does not interact with other Atg proteins or the vacuole (Shintani et al., 2002; Watanabe et al., 2010). To prevent vacuolar recruitment of Vac8, we used a truncation mutant lacking the N-terminal 18 amino acids (Vac8ΔN), which remains cytosolic due to the lack of acylation (Fig. 5A). Vac8ΔN was fused to the minimal reported Ape1-interacting region of Atg19, amino acids 152–191 (t-Vac8ΔN). This 40 amino acid-long peptide of Atg19 is sufficient for Ape1 binding but lacks the Atg11-, Ams1- and Atg8-binding regions (Shintani et al., 2002). Therefore, the fusion construct t-Vac8ΔN should be recruited to cytosolic Ape1 oligomers in *atg19*Δ cells (Fig. 5B). Indeed, t-Vac8ΔN–GFP colocalized with BFP–Ape1 in *atg19*Δ*vac8*Δ cells, whereas Vac8ΔN–GFP showed dispersed cytoplasmic signal. t-Vac8ΔN–GFP formed a distinct punctum in the cell, which was lost in the absence of Ape1, validating our experimental setup (Fig. 5C).

Vac8 has been reported to directly interact with the early PAS component Atg13 (Scott et al., 2000), therefore we asked whether ectopically localized Vac8 was still able to recruit Atg13. Indeed, t-Vac8ΔN–BFP formed distinct puncta and was able to recruit Atg13–GFP, which was not observed when expressing cytosolic Vac8ΔN–BFP, suggesting that ectopically localized Vac8 is able to recruit the PAS component Atg13 (Fig. 5D,E).

### Vac8 mediates PAS anchoring via the C-terminus of Atg13

**Fig. 6.**
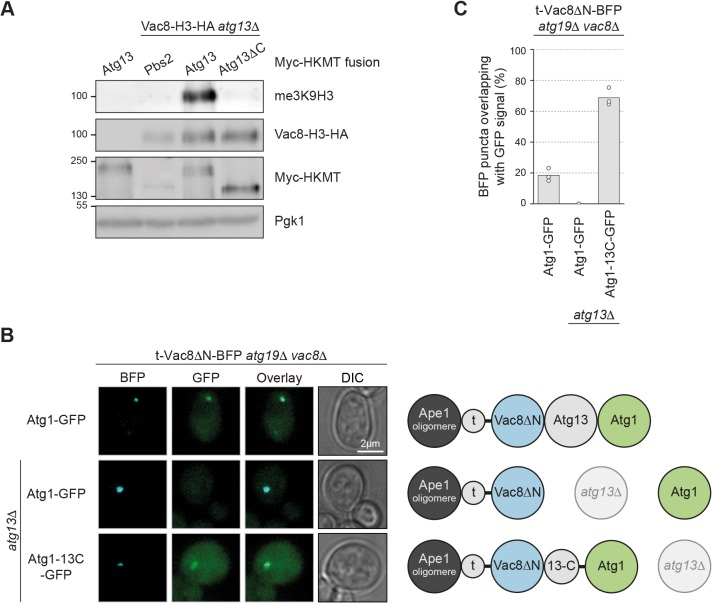
**Vac8 mediates PAS anchoring via the C-terminus of Atg13.** (A) Cells carrying a plasmid expressing Myc–HKMT-tagged Atg13, Atg13ΔC (Atg13^1-520^) or as a control Pbs2, and either endogenously expressed H3–HA-tagged Vac8 or untagged Vac8 were grown to mid-log phase in SD medium and treated for 4 h with 220 nM rapamycin. Cell extracts were prepared by TCA precipitation. The Vac8–H3–HA tri-methylation signal was assessed by anti-me3K9H3 western blotting. Vac8–H3–HA expression levels, Myc–HKMT-tagged Atg13 or Pbs2 expression levels, and loading were monitored by anti-HA, anti-Myc and anti-Pgk1 western blotting, respectively. One representative experiment out of three is shown. (B) *atg19*Δ*vac8*Δ or *atg13*Δ*atg19*Δ*vac8*Δ cells containing endogenously expressed Atg1–GFP or Atg1–Atg13C^567-738^–GFP were transformed with a plasmid expressing t-Vac8ΔN–BFP. Cells were grown to mid-log phase in SD medium and starved for 1 h in SD-N medium. Representative fluorescence images are shown. t, tether; DIC, differential interference contrast. (C) Quantification of B. The percentage of BFP puncta overlapping with GFP signal was quantified in three independent experiments; the values of each replicate (circles) and the mean (bars) were plotted.

Vac8 interacts with the C-terminus of Atg13 in a yeast two-hybrid assay (Scott et al., 2000). We re-evaluated this interaction *in vivo* by performing a methylation tracking (M-track) proximity assay (Fig. 1I). We fused Atg13 and Atg13ΔC to the HKMT domain, and Vac8 to the H3 tag. As expected, we observed methylation of Vac8–H3 with Atg13–HKMT, but only background levels of methylation with Atg13ΔC–HKMT (Fig. 6A), confirming that the Atg13 C-terminus is required for the interaction between Vac8 and Atg13 *in vivo*.

Next, we asked whether ectopic Vac8 is able to recruit additional PAS proteins. Atg1 is a PAS component and its PAS recruitment depends on Atg13 (Suzuki et al., 2007). We reasoned that if Vac8 acts as a PAS anchor by interacting with Atg13, then Atg1 should also be recruited to ectopic Vac8, but in an Atg13-dependent manner. Indeed, Atg1–GFP colocalized with t-Vac8ΔN–BFP puncta in *atg19*Δ*vac8*Δ cells, supporting the idea that other PAS components are also recruited to ectopic Vac8. This colocalization and Atg1–GFP punctum formation were lost in the absence of Atg13 (Fig. 6B,C). When fusing the C-terminal 172 amino acid Vac8-binding region of Atg13 to Atg1 (Atg1-13C), the localization of Atg1 to ectopic Vac8 was restored. Overall, these data suggest that Vac8 interacts with the PAS via the C-terminus of Atg13.

### The C-terminus of Atg13 links the PAS to the vacuolar tether Vac8

**Fig. 7.**
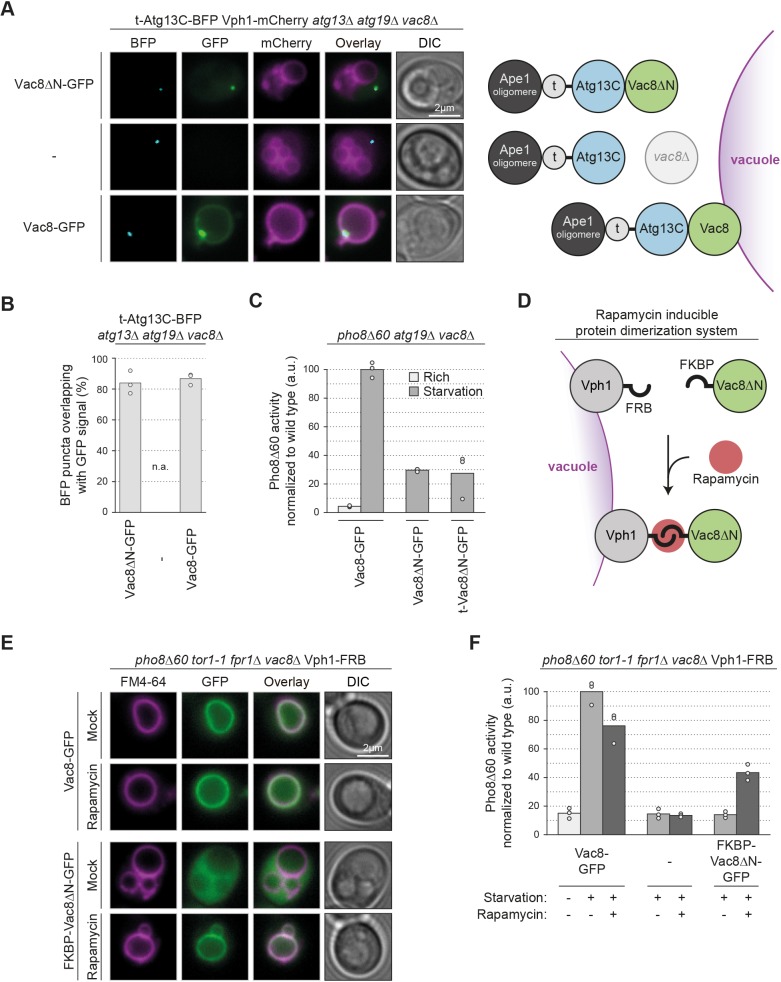
**Vac8-mediated vacuolar localization of the PAS is required for bulk autophagy.** (A) Vph1–mCherry *atg13*Δ*atg19*Δ*vac8*Δ cells carrying a plasmid expressing t-Atg13C–BFP and either Vac8ΔN–GFP, Vac8–GFP or an empty plasmid were grown to mid-log phase in SD medium and starved for 1 h in SD-N medium. Representative fluorescence images are shown (see also Fig. S4A). (B) Quantification of A. The percentage of BFP puncta overlapping with GFP signal was quantified in three independent experiments; the values of each replicate (circles) and the mean (bars) were plotted. (C) *pho8*Δ*60 atg19*Δ *vac8*Δ cells transformed with a plasmid expressing Vac8–GFP, Vac8ΔN–GFP or t-Vac8ΔN–GFP were grown to mid-log phase in SD medium and starved for 4 h in SD-N medium where indicated. Pho8Δ60 alkaline phosphatase activity was measured in three independent experiments. The values of each replicate (circles) and the mean (bars) were plotted. All values were normalized to the mean Pho8Δ60 alkaline phosphatase activity of cells expressing Vac8–GFP. (D) Schematic of experimental setup to inducibly tether Vac8 to the vacuole. The vacuolar transmembrane protein Vph1 is fused to an FRB domain and cytosolic Vac8ΔN to an FKBP domain. Upon addition of rapamycin the FRB and FKBP domains dimerize, which results in rapid and stable recruitment of Vac8ΔN to the vacuole. (E) *pho8*Δ*60 tor1-1 fpr1*Δ *vac8*Δ Vph1–FRB cells transformed with a plasmid expressing Vac8–GFP or FKBP–Vac8ΔN–GFP were grown to mid-log phase in SD medium, labelled with FM4-64 and starved for 1 h in SD-N medium with or without 1.5 µM rapamycin where indicated. Representative fluorescence images are shown. (F) *pho8*Δ*60 tor1-1 fpr1*Δ *vac8*Δ Vph1–FRB cells transformed with a plasmid expressing Vac8–GFP or FKBP–Vac8ΔN-GFP or an empty vector were grown to mid-log phase in SD medium, starved for 4 h in SD-N medium with or without 1.5 µM rapamycin where indicated. Pho8Δ60 alkaline phosphatase activity was measured in three independent experiments. The values of each replicate (circles) and the mean (bars) were plotted. All values were normalized to the mean Pho8Δ60 alkaline phosphatase activity of cells expressing Vac8–GFP. t, tether; DIC, differential interference contrast; a.u., arbitrary units.

If the C-terminus of Atg13 links the PAS to Vac8 on the vacuole, then ectopically localized Atg13C should also be sufficient to recruit Vac8ΔN. To test this scenario, we fused the Ape1-interacting region of Atg19 to the C-terminus of Atg13 (t-Atg13C). Indeed, t-Atg13C–BFP was sufficient to recruit Vac8ΔN–GFP to cytosolic Ape1 structures, visible as colocalizing fluorescence puncta in the cell. As expected, these puncta remained largely cytosolic in *atg13*Δ*atg19*Δ*vac8*Δ cells or in cells containing cytosolic Vac8ΔN–GFP (Fig. 7A,B; Fig. S4A). However, in the presence of wild-type vacuole-localized Vac8–GFP, the t-Atg13C fluorescent puncta were efficiently recruited to the vacuole. Interestingly, this also resulted in an enrichment of Vac8 at the vacuolar contact site of the t-Atg13C–BFP punctum (Fig. S4A,B). Together, these findings further substantiate that Vac8 anchors the PAS via its interaction with the C-terminus of Atg13, and suggest that Atg13 provides a link between the PAS and the vacuolar tether Vac8.

### Vac8-mediated PAS localization to the vacuole is required for autophagy function

Ectopic t-Vac8ΔN was able to recruit PAS components (Fig. 6C). To test whether this ectopic PAS localization away from the vacuole is sufficient to rescue the *vac8*Δ bulk autophagy defect, we monitored Pho8Δ60 activity. Both cells expressing Vac8ΔN and t-Vac8ΔN were strongly impaired in Pho8Δ60 activity, suggesting that ectopic PAS localization is not sufficient to drive autophagy (Fig. 7C).

To investigate whether vacuolar localization of Vac8, and thus PAS–vacuole contacts, are required for autophagosome formation, we artificially recruited cytosolic Vac8ΔN to vacuoles and asked whether this restores bulk autophagy function. We employed the inducible FKBP/FRB/rapamycin tethering system (FKBP, 12-kDa FK506 binding protein; FRB, FKBP-rapamycin binding domain). The method is based on rapamycin-induced dimerization of two proteins of interest tagged with FKBP and FRB, respectively (Choi et al., 1996). These experiments were performed in a *tor1-1* mutant background, in which a mutation in the FRB region of Tor1 prevents FKBP–rapamycin binding, thereby rendering *tor1-1* mutant cells resistant to rapamycin. In addition, FPR1 (FK506-sensitive proline rotamase, the yeast homolog of FKBP) was deleted to prevent competition for rapamycin binding (Heitman et al., 1991).

We used *pho8*Δ*60 tor1-1 fpr1*Δ *vac8*Δ Vph1-FRB cells expressing FKBP–Vac8ΔN. Rapamycin addition resulted in the tethering of FKBP–Vac8ΔN to Vph1–FRB (Fig. 7D,E). To test whether this is sufficient to restore the function of cytosolic Vac8ΔN, we monitored Pho8Δ60 activity. In the absence of rapamycin, cells expressing FKBP–Vac8ΔN were impaired in Pho8Δ60 activity. However, upon rapamycin treatment Pho8Δ60 activity was restored to 50% of wild-type levels (Fig. 7F). Similar results were obtained for Vac8ΔN tethered constitutively to the vacuole by fusing it directly to Vph1 (Fig. S4C). These findings further support the hypothesis that that PAS–vacuole contacts are required for efficient autophagosome formation.

The middle domain of Atg13 (amino acids 269–520, Atg13M) contains binding regions for Atg1 and Atg17 (Fujioka et al., 2014); thus, we speculated that vacuolar localized Atg13M might be sufficient to recruit the PAS to the vacuole and to promote bulk autophagy independently of Vac8. To test this possibility, we fused Atg13M to the vacuolar protein Vph1 and expressed it in addition to endogenous Atg13 in wild-type and *vac8*Δ cells (Fig. S4D). However, in a Pho8Δ60 assay, the Vph1–Atg13M fusion protein failed to bypass the requirement for Vac8 in bulk autophagy (Fig. S4C). This could be due to technical reasons or it could indicate that Vac8 not only anchors the PAS at the vacuole but also serves additional functions in bulk autophagy.

In summary, our findings suggest that Vac8 promotes the formation of autophagosomes and autophagosome–vacuole fusion by tethering the PAS and the different autophagosomal intermediates via the C-terminus of Atg13 to the vacuole.

## DISCUSSION

**Fig. 8.**
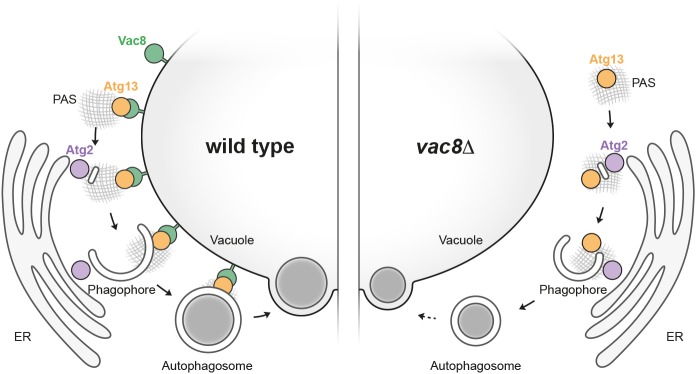
**Vac8 confines autophagosome formation between the vacuole and the ER by tethering the PAS to the vacuole.** Vac8 tethers the early PAS to the vacuole via interaction with Atg13. Maturation of the PAS by recruitment of downstream Atg factors, including Atg2, results in establishment of an ER–PAS–vacuole connection. Consequently, autophagosome formation takes place in a confined space between the ER and the vacuole. This allows autophagosome formation to be spatially coordinated with autophagosome–vacuole fusion. In a *VAC8* deletion mutant, the connection between PAS and vacuole is lost and the site of autophagosome formation remains only at the ER. As a consequence, PAS initiation is decreased, autophagosomes form with a reduced size and are ultimately released into the cytosol, which makes the autophagosome–vacuole fusion process inefficient.

Our work shows that Vac8 plays a direct and important role in bulk autophagy. Vac8 is not only required for efficient initiation of PAS formation but also for formation and subsequent fusion of autophagosomes with the vacuole. Based on the Vac8-dependent association of Atg8-positive structures with the vacuole, we propose that Vac8 anchors the early PAS to the vacuole and maintains this connection throughout the biogenesis of the autophagosome. In this way, Vac8 coordinates the site of autophagosome formation with vacuole fusion and improves the efficiency of bulk autophagy by localizing autophagosome formation within the cell (Fig. 8).

Although decreased autophagic function had been observed in *vac8*Δ mutants (Cheong et al., 2005; Scott et al., 2000), the molecular function of Vac8 in autophagy had remained unknown. Deletion of *VAC8* was found to reduce the degradation of autophagic cargo in the vacuole, the end point of the pathway, and this was interpreted as a severe bulk autophagy flux defect (Cheong et al., 2005; Scott et al., 2000). However, the assay used relies on vacuolar function. Since Vac8 is also required for efficient homotypic vacuole fusion, it had remained unclear whether Vac8 actually plays a direct role in autophagy or whether the observed autophagy defect in *vac8*Δ mutants rather stemmed from a general defect in vacuolar function. Our work reveals a direct function for Vac8 in autophagy. It acts early in autophagy by tethering the PAS and forming autophagosomes to the vacuole. Vac8 itself is stably anchored to the vacuole by N-terminal acylation and its vacuolar localization has been reported to be important for establishing nucleus–vacuole, vacuole–vacuole and vacuole–cytoskeleton connections. We show that N-terminal acylation of Vac8 is also essential for its function in autophagy, which is in line with Vac8 serving as a vacuolar tether of the PAS.

PAS anchoring is mediated via Vac8 binding to the C-terminus of Atg13. Atg13 in turn then recruits further Atg proteins to assemble the PAS at the vacuole. Interestingly, clustering of Atg13 C-terminus on ectopic cytosolic Ape1 results in the recruitment of this structure to the vacuole, but also the enrichment of vacuolar Vac8 at the Ape1–Atg13C–vacuole contact site (Fig. 7A; Fig. S4A,B). This enrichment of Vac8 might be caused by a high local concentration of t-Atg13C on Ape1. Similarly, Vac8 has been observed to be enriched at nucleus–vacuole junctions, suggesting that it has the ability to locally cluster on the vacuolar surface where needed. The role of this local clustering of Vac8 and whether this also happens during native PAS assembly is an interesting subject for future studies.

Ectopic Vac8ΔN clustered on a cytosolic oligomer is able to assemble PAS components, but it is not sufficient to restore bulk autophagy function (Figs 6B and 7C). However, vacuolar tethering of Vac8ΔN partially rescued the bulk autophagy defect of *vac8*Δ mutants (Fig. 7F; Fig. S4C), underlining the necessity for the vacuolar localization of the PAS. Vac8ΔN artificially tethered to the vacuole lacks N-terminal myristoylation and palmitoylation. Interestingly, it was previously reported that palmitoylation of Vac8 might have a direct function in mediating vacuole–vacuole fusion and in vacuolar inheritance (Subramanian et al., 2006). Thus, it could be speculated that palmitoylation of Vac8 also affects its function in bulk autophagy. Attempts to fully bypass Vac8 function by tethering the PAS to the vacuole via a vacuolar localized middle domain of Atg13 failed (Fig. S4C). This could be due to technical reasons, for example, that the bypass system fails to appropriately recruit PAS factors to the vacuole. Alternatively, this result could indicate that Vac8 not only anchors the PAS to the vacuole via interaction with C-terminus of Atg13 but that it is also required for additional functions in bulk autophagy, such as fusion of autophagosomes with the vacuole. Further studies will be required to fully understand the mechanistic details of the PAS–autophagosome–vacuole connection.

The growing phagophore has been observed to be positioned adjacent to the vacuole and, concurrently, also have one edge connected to the ER, with Atg2 being required for maintaining the ER connection (Gómez-Sánchez et al., 2018; Graef et al., 2013; Suzuki et al., 2013). Our results therefore suggest that Atg2 and Vac8 are key determinants in establishing an ER–PAS-vacuole connection, thereby localizing the formation of autophagosomes between the ER and the vacuole. This organization principle allows autophagosome formation to be spatially coordinated with vacuole fusion. The endosomal Rab family protein Vps21 is required for sealing autophagosomes, and indeed a *VPS21* deletion mutant displays accumulation of unsealed autophagosomes that remain attached to the vacuole (Zhou et al., 2017). This further supports our model where autophagosomes are anchored to the vacuole by Vac8 throughout the whole autophagosome formation process until they fuse with the vacuole (Fig. 8).

In *VAC8* deletion mutants, autophagosomes form adjacent to the ER without being stably tethered to the vacuole. Loss of this vacuolar connection results in autophagosomes accumulating in the cytosol of *vac8*Δ cells (Figs 2F and 3D), suggesting that complete autophagosomes are released from the ER but fail to establish a connection to the vacuole. Nevertheless, autophagosomes ultimately can fuse with the vacuole, although with a substantial temporal delay. A correct topological orientation of the phagophore relative to the ER is important for autophagosome formation (Gómez-Sánchez et al., 2018). So far, however, the relevance of the vacuolar connection for phagophore expansion has remained unclear. Bulk autophagosomes formed in *vac8*Δ mutants are smaller than in wild-type cells, indicating that the PAS–vacuole connection also plays an important structural role for normal autophagosome formation. In mammals, the omegasome, a specialized subdomain of the ER, has been reported to serve as a cradle for the formation of the phagophore (Hayashi-Nishino et al., 2009; Kast and Dominguez, 2017). It is conceivable that the ER–vacuole connection might serve an analogous role in yeast. The precise molecular reason for the bulk autophagosome size defect caused by deletion of *VAC8* remains to be determined.

In mammalian cells, autophagosomes are made throughout the cytosol, forming in specialized subdomains of the ER (Karanasios et al., 2016; Nakamura and Yoshimori, 2017). Endosomes and lysosomes, by contrast, are predominantly found in the perinuclear region. As a result, completed autophagosomes need to be actively transported into the perinuclear region to allow effective fusion with lytic organelles. This transport is mediated by the microtubular network (Mackeh et al., 2013). In yeast, however, the cytoskeleton is dispensable for bulk autophagy (Kirisako et al., 1999; Reggiori et al., 2005). Our study sheds light on the reasons for this fundamental difference among eukaryotic species. Bulk autophagosome formation in yeast is initiated at the vacuole and apparently this connection is maintained while the autophagosome is forming and, most likely, until the point of fusion with the vacuole. Hence, active transport of mature autophagosomes to the lytic organelle is superfluous. The observation that *vac8*Δ cells accumulate cytosolic autophagosomes underpins the significance of a stable autophagosome–vacuole connection in yeast. This difference in autophagosome formation might stem from the apparent difference in the size of the lytic compartment and its distribution in the cell. Whereas yeast cells contain one to only a few large vacuoles that take up a substantial part of the cell, mammalian cells contain many small lysosomes that are enriched in the perinuclear region.

In contrast to bulk autophagy, selective autophagy requires the actin cytoskeleton in yeast (Reggiori et al., 2005). Future studies are needed to better understand the functional relevance of the cytoskeleton and the vacuolar connection of the PAS during selective autophagy in yeast.

## MATERIALS AND METHODS

### Yeast strains

Yeast strains are listed in Table S1 (Bas et al., 2018; Kijanska et al., 2010; Robinson et al., 1988; Torggler et al., 2016). Yeast strains and plasmids used in each figure are listed in Table S3. Genomic insertions (tagging) were performed according to Janke et al. (2004) or Longtine et al. (1998); multiple deletions or mutations were generated by PCR knockout or mating and dissection.

### Plasmid construction

Plasmids are listed in Table S2 (Mari et al., 2010; Pfaffenwimmer et al., 2014; Sikorski and Hieter, 1989; Torggler et al., 2016; Welter et al., 2010). For pMS5 (Vac8–GFP), Vac8 with its endogenous promoter (626 bases) was amplified from genomic DNA and ligated into pRS415 vector using NotI/PstI restriction sites; GFP was PCR amplified and subcloned via PstI/SalI. Vac8–GFP mutants (pMS13, pMS130, pMS131 and pDH1) were generated by ‘round the horn’ PCR using pMS5 (Vac8–GFP) as a template and primers containing the respective mutations. To create pDH2 [FKBP-Vac8(19-578)–GFP], FKBP was PCR amplified and subcloned into pDH1 via SpeI/SbfI. For pDH8 [Atg19(152-191)–Vac8(19-578)–GFP], the Ape1-binding region of Atg19 (amino acids 152–191) was PCR amplified from genomic DNA and subcloned into pDH2 via SpeI/BamHI. pDH10 [Atg19(152-191)–Vac8(19-578)–GFP], pDH11 [Vac8(19-578)–GFP] and pDH12 [Vac8–GFP] were generated by exchanging the pRS415 vector to pRS413 via SacI/SalI restriction cloning. For pDH9 [Atg19(152-191)–Vac8(19-578)–mTagBFP2] and pDH13 [Vac8(19-578)–mTagBFP2], the GFP tag of pDH8 and pDH1, respectively, was replaced with mTagBFP2 via PstI/SalI; mTagBFP2 was PCR amplified. For pDH14 [Atg19(152-191)-Atg13(567-738)-mTagBFP2], the C-terminal region of Atg13 (amino acids 567–738) was PCR amplified from genomic DNA and ligated into pDH9 via BamHI/PstI. For pDH33, the *VAC8* promoter was cut out from pDH1 using NotI/SpeI, Vph1 was PCR amplified from genomic DNA and cut with SpeI/BamHI, mScarlet was PCR amplified with an N-terminal and C-terminal AGSAAGS linker and cut with BamHI/SalI, and all were ligated into a pRS416 vector. For pDH32, the middle region of Atg13 (amino acids 269–520) was PCR amplified from genomic DNA and ligated into pDH33 using SbfI/SalI restriction sites. For pDH39, Vac8 (amino acids 19–578) was PCR amplified from genomic DNA and ligated into pDH33 using the SbfI/SalI restriction sites. For pDH41, the *ATG13* endogenous promoter (698 bases) was PCR amplified from genomic DNA and ligated into pDH32 using NotI/SpeI restriction sites. For pAB19, Pbs2 with its endogenous promoter (296 bases) was amplified from genomic DNA and ligated into pCK903 vector (Brezovich et al., 2015) using the NotI/SbfI restriction sites. For pRT118, Atg13 with its endogenous promoter (698 bases) was amplified from genomic DNA and ligated into pCK903 vector using the NotI/SbfI restriction sites. For pRT119, Atg13 (amino acids 1–520) with its endogenous promoter (698 bases) was amplified from genomic DNA and ligated into pCK903 vector using NotI/SbfI restriction sites. For pAC160, Nvj1 with its endogenous promoter (700 bases) was amplified from genomic DNA and ligated into pCK903 vector using NotI/SbfI restriction sites.

### Antibodies

The following primary antibodies were used in this study: rabbit polyclonal PAP antibody (1:5000, P1291, Sigma-Aldrich), polyclonal rabbit anti-Ape1 antibody (1:20,000; Torggler et al., 2016), mouse monoclonal anti-Pgk1 antibody (1:10,000; 22C5D8, Invitrogen), mouse monoclonal anti-GFP antibody (1:100; 2B6, Max F. Perutz Laboratories, Monoclonal Antibody Facility), rabbit monoclonal anti-HA antibody (1:1000; EPR4095, Abcam), mouse monoclonal anti-me3K9H3 antibody (1:2000; 6F12-H4, Novus Biochemicals) and mouse monoclonal anti-Myc antibody (used 1:5000) (4A6, Millipore).

### Growth conditions

Yeast cells were grown in synthetic medium (SD, 0.17% yeast nitrogen base, 0.5% ammonium sulfate, 2% glucose and amino acids as required) or rich medium (YPD, 1% yeast extract, 2% peptone and 2% glucose) to mid-log phase. To induce bulk autophagy, cells were washed and resuspended in nitrogen starvation medium (SD-N: 0.17% yeast nitrogen base without amino acids, 2% glucose) or treated with 220 nM rapamycin for the indicated time to induce autophagy. Yeast liquid cultures were incubated with shaking (200 rpm) at 30°C.

### Serial dilution spot assay

Yeast cell cultures were grown to mid-log phase in YPD medium, shifted to SD-N medium containing 10 µg/ml tetracycline, diluted to an optical density at 600 nm (OD_600_) of 0.1 and incubated at 30°C. For analysing cell viability, a 7-fold serial dilution series was prepared by diluting cell cultures in SD-N medium, and 5 µl of each dilution was spotted onto YPD plates. Pictures were taken after 48 h incubation at 30°C.

### Standard biochemical assays

For trichloroacetic acid (TCA) extract preparation, 1.5 ml of yeast cell culture was precipitated with 7% TCA and incubated for 10 min on ice. Precipitated proteins were pelleted at 13,000 ***g*** for 5 min at 4°C, washed with 1 ml acetone, air-dried, resuspended in urea loading buffer (120 mM Tris-HCl pH 6.8, 5% glycerol, 8 M urea, 143 mM β-mercaptoethanol, 8% SDS) and boiled before being loaded onto SDS-PAGE gels. Protein extracts were transferred onto nitrocellulose membranes and proteins were detected by immunoblotting, using the ECL detection system.

For preparation of freezer milled yeast powder, cells were harvested by centrifugation (3000 ***g***, 10 min, room temperature), washed with 1× PBS containing 2% glucose and resuspended in 1 µl/OD_600_ unit (one OD_600_ unit corresponds to 1 ml yeast culture with an OD_600_ of 1) IP buffer [1× PBS, 10% glycerol, 0.5% Tween-20, 1 mM NaF, 1 mM PMSF, 20 mM β-glycerophosphate, 1 mM Na_3_VO_4_ and cOmplete™ protease inhibitor cocktail (Roche)]. Cells were milled in a cryogenic grinder (SPEX Freezer Mill 6875, SPEX SamplePrep), using five rounds of 3 min breakage at 15 cycles per second and 2 min cooling. Yeast powder was stored at −80°C.

### Atg1 kinase assay

0.8 g freezer milled yeast powder was thawed on ice and 500 µl IP buffer was added (see section ‘Standard biochemical assays’). Extract was cleared by centrifugation (twice for 10 min, 5000 ***g***, 4°C) and protein concentration was adjusted to 20–25 µg/µl in 700 µl IP buffer. Extract was incubated with 10 µl IgG-coupled Dynabeads (Thermo Fisher Scientific) on an end-over-end rotating wheel for 1 h at 4°C. Beads were washed three times for 5 min in IP buffer and afterwards split for western blotting (5 µl beads) and the kinase assay (5 µl beads). Beads for western blotting were eluted in 10 µl urea loading buffer and blotted for the ProteinA tag. Beads for the kinase assay were washed in 1× kinase buffer (20 mM HEPES pH 7.4, 150 mM potassium acetate, 10 mM magnesium acetate, 0.5 mM EGTA, 5 mM NaCl) and resuspended in 11 µl kinase mix {1× kinase buffer, 10 mM Na_3_VO_4_, 3 µg GST-tagged Atg19 C-terminal fragment used as substrate (Pfaffenwimmer et al., 2014), 2.5 µCi γ-[^32^P]ATP}. After 20 min at 30°C, beads and supernatant were separated and mixed with urea loading buffer. Samples were analysed by SDS-PAGE and phosphor-imaging.

### Pho8Δ60 assay

25 OD_600_ units of yeast culture were harvested by centrifugation (3000 ***g***, 5 min, room temperature). Pellets were washed in 1 ml distilled H_2_O, followed by centrifugation (2000 ***g***, 5 min, 4°C) and resuspension of the pellet in 2 ml ice-cold 0.85% NaCl containing 1 mM PMSF. After another centrifugation step (2000 ***g***, 5 min, 4°C), pellets were resuspended in 16 µl/OD_600_ unit lysis buffer [20 mM PIPES pH 6.8, 0.5% Triton X-100, 50 mM KCl, 100 mM potassium acetate, 10 mM MgSO_4_, 10 µM ZnSO_4_, 1 mM PMSF, cOmplete™ protease inhibitor cocktail (Roche)]. Cells were lysed by bead beating, and extracts were cleared by centrifugation (16,000 ***g***, 5 min, 4°C). Protein concentration of the supernatant was adjusted to 50 µg in 100 µl lysis buffer. 400 µl reaction buffer (0.4% Triton X-100, 10 mM MgSO_4_, 10 µM ZnSO_4_, and 250 mM Tris-HCl pH 8.5) containing 6.25 mM α-naphthylphosphate (Sigma-Aldrich) was added to enzymatic reactions, or only reaction buffer was added to control reactions. Reactions were incubated at 37°C for 10 min and stopped by adding 500 µl stop buffer (1 M glycine pH 11). Fluorescence was measured using 345 nm for excitation and 472 nm for emission.

For each experiment, three independent replicates were performed. Normalized Pho8Δ60 activity was calculated as follows: a standard curve was generated by either using a dilution series of the product (1-naphtol, Sigma-Aldrich) or a reaction time course series of the starvation-induced wild-type sample (20, 10, 5, 2.5 and 0 min). Least squares linear regression was performed using the standard curve, which was then used to calculate the relative abundance values of the samples. Replicates were normalized to each other by subtracting the averaged differences of the sample means. The replicate mean of the starvation-induced wild-type sample was set to 100.

### M-track *in vivo* protein–protein proximity assay

M-track assays were performed as described previously (Brezovich et al., 2015; Zuzuarregui et al., 2012). Cell extracts were prepared by TCA precipitation and analysed by western blotting.

### *In vitro* fusion assay

Fusion assays were performed according to Bas et al. (2018) with some adaptations. Vacuoles were isolated from Vph1–mCherry *atg15*Δ *pep4*Δ cells. Cells were lysed with DEAE-dextran according to Gao et al. (2018) and Haas (1995) and vacuoles purified on a Ficoll gradient at the 0–4% interface. Vacuoles were incubated with autophagosomal fractions prepared from GFP–Atg8 *pep4*Δ*vam3*Δ cells and successful fusion was monitored by fluorescence microscopy. ATP was depleted by apyrase treatment, which converts ATP into AMP.

### Quantitative live-cell imaging

Live-cell imaging was performed at room temperature using an AxioObserver Z1 inverted microscope (ZEISS) equipped with an α-Plan Apochromat 100× oil/1.46 NA differential interference contrast RMS objective, a PCO 1600 camera, and a Lumencor SOLA 6-LCR-SB light source with VisiView software (Visitron Systems) (Figs 1E,F, 2A–F, 5A,C–E, 6B,C and 7A,B,E; Fig. S4A,B,D) or with a DeltaVision Elite RT microscope system (GE Healthcare, Applied Precision), equipped with a UPLASPO 100× oil/1.40 NA objective, a pco.edge 5.5 sCMOS camera (PCO) and a seven-colour InsightSSI solid-state illumination system (GE Healthcare, Applied Precision) (Fig. 4A,C–E; Fig. S3B,C).

Quantitative analysis of PASs, forming autophagosomes or completed autophagosomes was performed by counting the number of GFP–Atg8, Atg1–GFP or Atg2–GFP puncta per cell. Images were generated by collecting a *z*-stack of 21 pictures with focal planes 0.20 μm or 0.25 µm apart, in order to cover the entire volume of a yeast cell. Background subtraction was performed using the rolling ball algorithm with a 7-pixel radius for the green channel (PAS or autophagosomes). The number of PASs, forming autophagosomes or completed autophagosomes per cell was counted blindly after randomizing image names. Image analysis was performed using FIJI (Schindelin et al., 2012) (Figs 1E,F and 2A–F).

Subcellular positioning of PASs, forming autophagosomes or complete autophagosomes was investigated by analysing the localization of mCherryV5–Atg8 puncta in regard to the ER, using the genomically tagged ER marker protein Sec63–GFP, and vacuoles labelled with CellTracker™ Blue 7-amino-4-chloromethylcoumarin (CMAC) dye (Thermo Fisher Scientific). Distribution of Vac8–GFP variants was analysed in regard to CMAC-stained vacuoles. Images were generated by collecting a *z*-stack of 22 pictures with focal planes 0.20 μm apart. Image deconvolution and analysis were performed using SoftWoRx (Applied Precision) and ImageJ software (Schneider et al., 2012) (Fig. 4A,C–E, Fig. S3B,C).

Recruitment of Vac8 (t-Vac8ΔN) artificially tethered to Ape1 was investigated by analysing the localization of Vac8–GFP signal in regard to mTagBFP2–Ape1 puncta. Colocalization of GFP-tagged proteins to Ape1 tethered t-Vac8ΔN–BFP was investigated by analysing the localization of GFP signal in regard to t-Vac8ΔN–BFP puncta. Images were generated on one focal plane. For quantification of BFP puncta overlapping with GFP signal, background subtraction was performed using the rolling ball algorithm with a 5-pixel radius for the green and blue channel. Quantification was performed blindly after randomizing image names. Image analysis was performed using FIJI (Schindelin et al., 2012) (Figs 5A,C–E and 6B,C).

Recruitment of Vac8 or Vac8ΔN to Ape1-tethered Atg13C (t-Atg13C) was investigated by analysing the localization of Vac8–GFP or Vac8ΔN–GFP signal in regard to t-Atg13C–BFP puncta. Subcellular positioning of Ape1-tethered Atg13C (t-Atg13C) was investigated by analysing the localization of t-Atg13C–BFP puncta in regard to vacuoles, using the genomically tagged vacuolar marker protein Vph1–4×mCherry. Images were generated by collecting a *z*-stack of 11 pictures with focal planes 0.25 μm apart. Image analysis was performed using FIJI (Schindelin et al., 2012) (Fig. 7A,B, Fig. S4A,B).

Distribution of FKBP–Vac8ΔN–GFP was analysed in regard to vacuoles stained with the FM™ 4-64 dye [*N*-(3-triethylammoniumpropyl)-4-(6-(4-(diethylamino) phenyl) hexatrienyl) pyridinium dibromide] dye (Thermo Fisher Scientific). Images were generated by collecting a *z*-stack of 11 pictures with focal planes 0.25 µm apart. Image analysis was performed using FIJI (Schindelin et al., 2012) (Fig. 7E).

Localization of Vph1–mScarlet fusion proteins was investigated by analysing the mScarlet signal. Images were generated by collecting a *z*-stack of 11 pictures with focal planes 0.25 µm apart. Image analysis was performed using FIJI (Schindelin et al., 2012) (Fig. S4D).

Subcellular positioning of the PASs or autophagosomes, or the number of PASs or autophagosomes per cell was determined by analysing ≥100 cells from three independent experiments. The number of Ape1-tethered t-Vac8ΔN–BFP or t-Atg13C–BFP puncta overlapping with the signal of GFP-tagged proteins was determined by analysing ≥75 BFP puncta from three independent experiments.

Images from each figure panel were taken with the same imaging setup and are shown with the same contrast settings. Single focal planes of representative images are shown.

### Electron microscopy

Fifteen OD_600_ units of cells were harvested by centrifugation (1800 ***g***, 5 min, room temperature). Cells were washed in distilled H_2_O and pelleted by centrifugation (1800 ***g***, 5 min, room temperature). Cells were resuspended in 3 ml of freshly prepared ice-cold 1.5% KMnO_4_ (Sigma) and transferred into two 1.5 ml microfuge tubes. After topping up the tube with the same solution to exclude air, samples were mixed on a rotatory wheel for 30 min at 4°C. After centrifugation (1400 ***g***, 3 min, 4°C), the 1.5% KMnO_4_ incubation was repeated once more before washing the pellets five times with 1 ml of H_2_O.

Permanganate-fixed cells were dehydrated stepwise with increasing concentrations of acetone (10%, 30%, 50%, 70%, 90%, 95% and three times 100%). Each incubation step was performed for 20 min at room temperature on a rotating wheel, and in between each step, cells were pelleted by centrifugation (1400 ***g***, 3 min, room temperature). After the last centrifugation, pellets were resuspended in 33% Spurr's resin in acetone and mixed on the same device for 1 h at room temperature. Cells were then harvested (7600 ***g***, 3 min, room temperature) and incubated in 100% freshly made Spurr's resin overnight on a rotating wheel at room temperature. This operation was repeated the following day, over the day, after centrifugation of the overnight incubation (9000 ***g***, 5 min, room temperature). The Spurr's resin mixture was prepared by mixing 10 g of 4-vinylcyclohexene dioxide (or ERL4206), 4 g of epichlorohydrin-polyglycol epoxy (DER) resin 736, 26 g of (2-nonen-1-yl)succinic anhydride (NSA) and 0.4 g of *N*,*N*-diethylethanolamine (all from Sigma). Afterwards, the cell and Spurr's mixture was transferred in size 00 embedding capsules (Electron Microscopy Science), and cells were pelleted by centrifugation (9000 ***g***, 5 min, room temperature). Embedding capsules were topped up with 100% Spurr's and were baked for a minimum of 3 days at 60°C.

Thin sections of ∼55 nm were cut using an ultramicrotome (Leica Microsystems). Cell sections were collected on formvar carbon-coated 100 meshes copper grids (EMS). Cell sections were stained with a filtered lead-citrate solution (80 mM lead nitrate, 120 mM sodium citrate pH 12) for 2 min at room temperature. Sections were viewed in a CM100bio TEM (FEI, Eindhoven, Netherlands).

## Supplementary Material

Supplementary information
