# Supplementary material for: Vac8 spatially confines autophagosome formation at the vacuole in S. cerevisiae
Source: J Cell Sci. 2019 Nov 14;132(22):jcs235002. doi: 10.1242/jcs.235002 (PMC6899017; doi:10.1242/jcs.235002)
Supplement: Supplementary information [file joces-132-235002-s1.pdf]

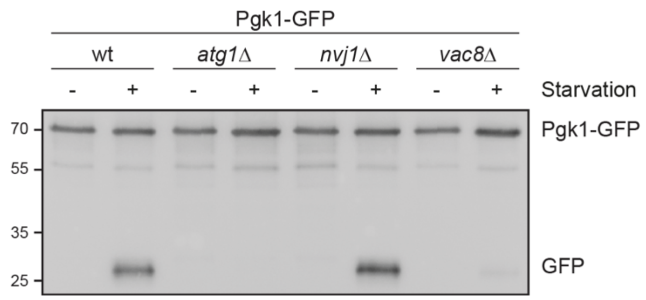

Figure S1: Nvj1 is not involved in bulk autophagy.
The indicated strains transformed with a plasmid expressing Pgk1-GFP were grown to mid-log phase in SD medium and starved for 4 hours in SD-N medium where indicated. Cell extracts were prepared by TCA precipitation. Pgk1-GFP cleavage was monitored by anti-GFP western blotting. One representative experiment out of two is shown.

Journal of Cell Science • Supplementary information

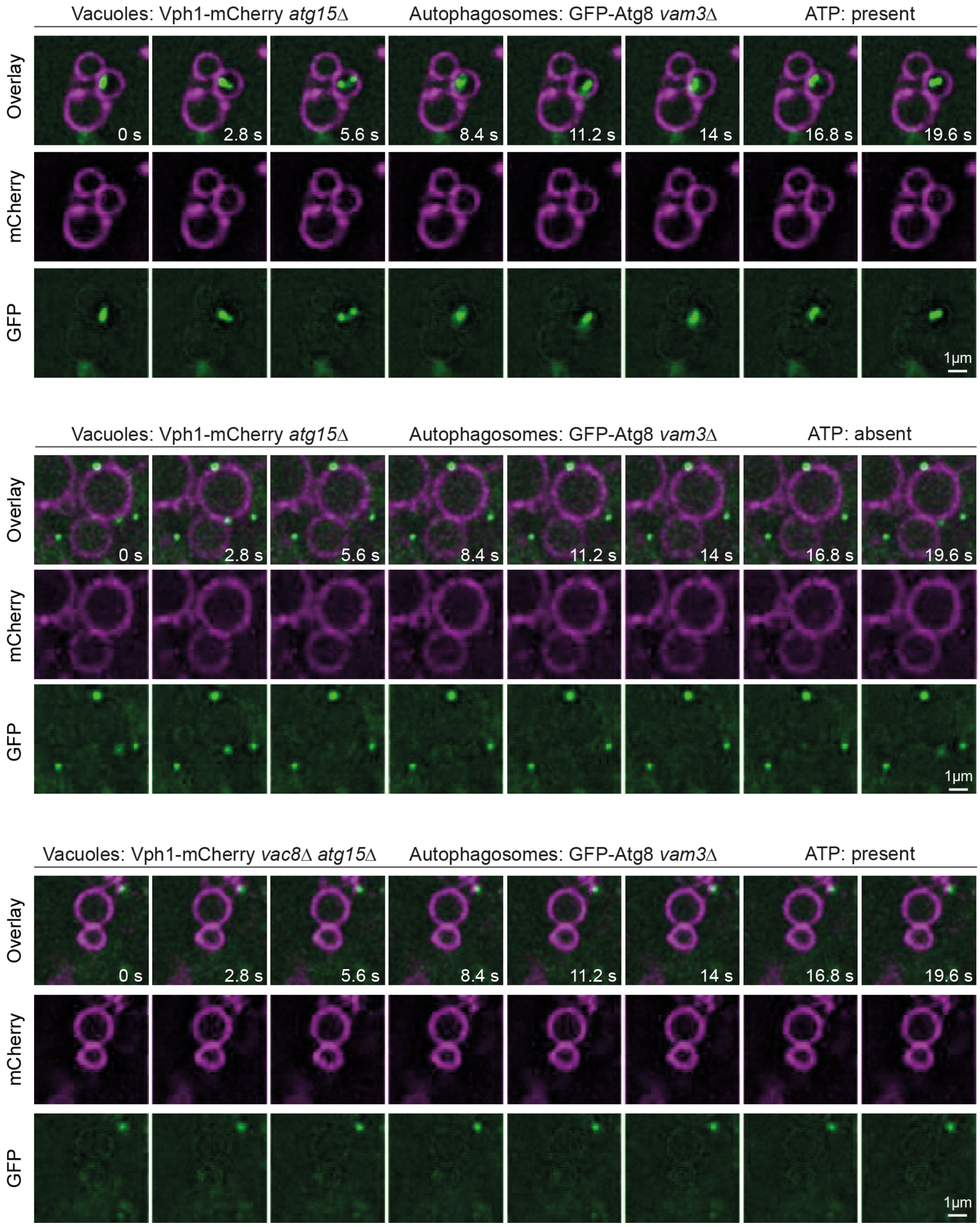

Figure S2: *vac8*Δ mutants are defective in autophagosome-vacuole fusion.
Vacuoles were isolated from Vph1-mCherry *atg15*Δ or Vph1-mCherry *atg15*Δ*vac8*Δ cells and incubated with autophagosomal fractions prepared from GFP-Atg8 *vam3*Δ cells and an energy regeneration system for 2 hours. Apyrase was added to deplete ATP where indicated. Fusion was monitored by fluorescence microscopy and judged by the appearance of a mobile green dot in the vacuole. Shown are stills of a 20 second time-lapse video (see also Figure 3E and 3F).

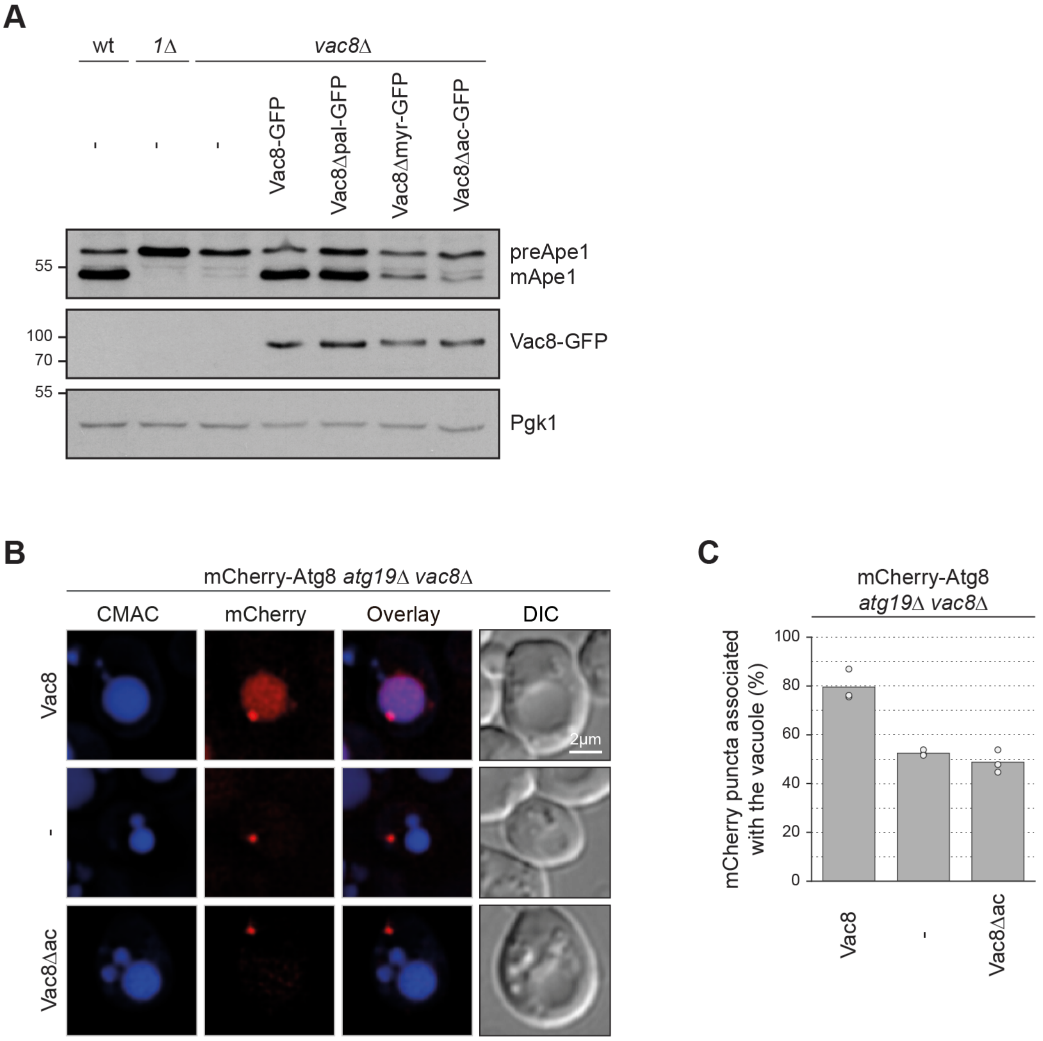

Figure S3: Acylation of Vac8 is required for its vacuolar localization and function in autophagy. (A) Indicated strains transformed with a plasmid expressing Vac8-GFP, Vac8Δpal-GFP (Vac8-C4G-C5T-C7S-GFP), Vac8Δmyr-GFP (Vac8-G2A-GFP) or Vac8Δac-GFP (Vac8-G2A-C4G-C5T-C7S-GFP) or an empty vector were grown to mid-log phase in SD medium and cell extracts were prepared by TCA precipitation. Ape1 processing was analyzed by anti-Ape1 western blotting. Vac8-GFP expression levels and loading were monitored by anti-GFP and anti-Pgk1 western blotting, respectively. One representative experiment out of two is shown.
(B) and (C) *atg19Δvac8Δ* cells carrying a plasmid expressing mCherry-V5-Atg8 and Vac8-GFP or Vac8Δac-GFP (Vac8-G2A-C4G-C5T-C7S-GFP) or an empty vector were grown to mid-log phase in SD medium, starved for 1 hour in SD-N medium and labelled with CMAC. Three independent experiments were performed. For each strain and replicate at least 100 cells were analyzed. Representative fluorescence images are shown in (B). The percentage of mCherry-Atg8 puncta associated with the vacuole (C) was quantified; the values of each replicate (circles) and the mean (bars) were plotted.
DIC, differential interference contrast.

Journal of Cell Science • Supplementary information

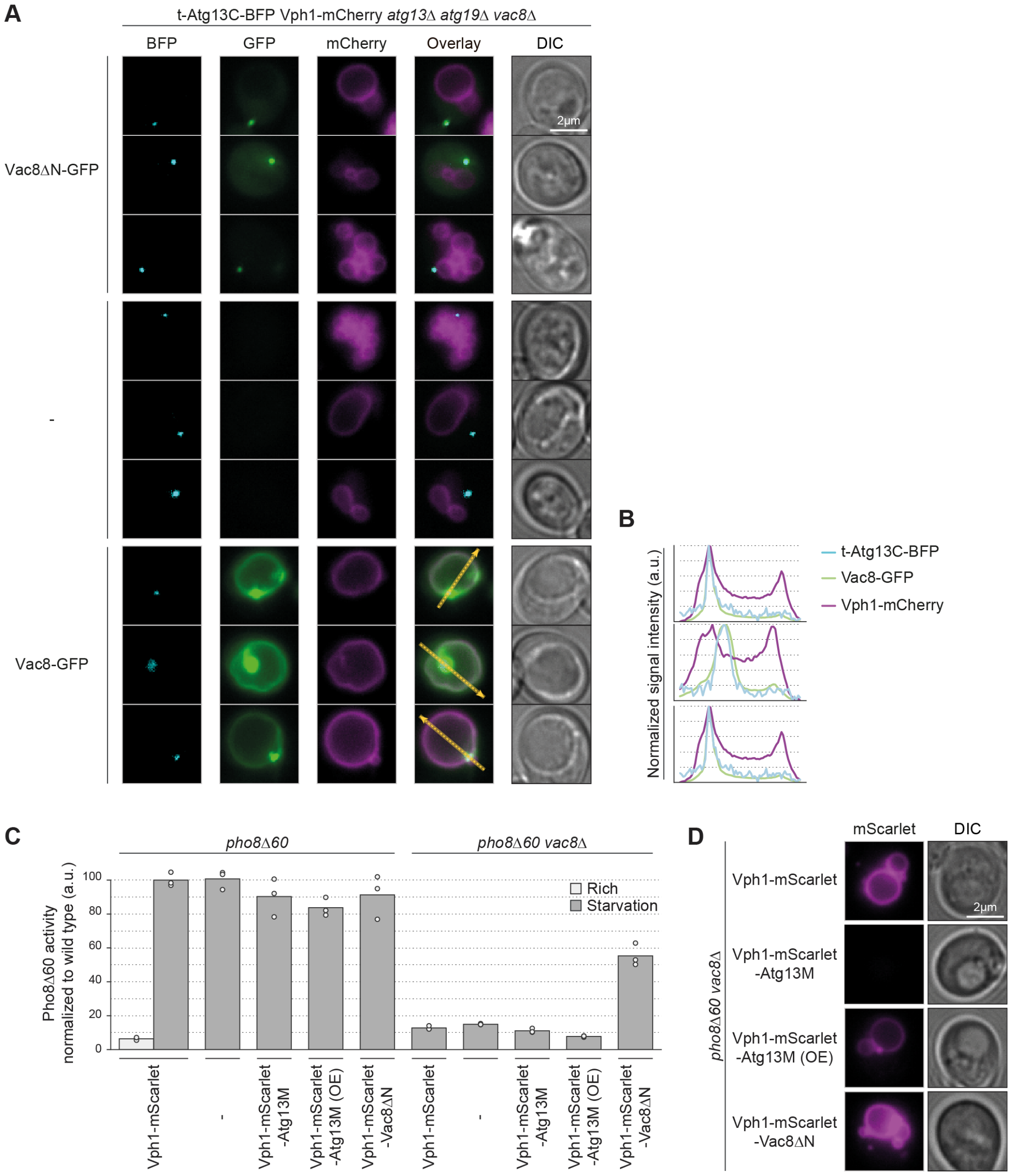

Figure S4: The C-terminus of Atg13 interacts with Vac8.

(A) Vph1-mCherry *atg13Δatg19Δvac8Δ* cells carrying a plasmid expressing t-Atg13C-BFP (Atg19$^{(152-191)}$-Atg13$^{(567-738)}$-BFP) and either Vac8ΔN-GFP (Vac8$^{(19-578)}$-GFP), Vac8-GFP or an empty plasmid were grown to mid-log phase in in SD medium and starved for 1 hour in SD-N medium. Representative fluorescence images are shown (see also Figure 7A and 7B).

(B) Profile plots showing Vac8-GFP signal enrichment along yellow lines in (A). For each channel intensities along the indicated lines were measured and normalized to the channel maximum and minimum values.

(C) Indicated strains transformed with a plasmid expressing Vph1-mScarlet, Vph1-mScarlet-Atg13M$^{(269-520)}$, overexpressed Vph1-mScarlet-Atg13M$^{(269-520)}$ (OE), Vph1-mScarlet-Vac8ΔN$^{(19-578)}$ or an empty vector were grown to mid-log phase in SD medium and starved for 4 hours in SD-N medium where indicated. Pho8Δ60 alkaline phosphatase activity was measured in three independent experiments. The values of each replicate (circles) and the mean (bars) were plotted. All values were normalized to the mean Pho8Δ60 alkaline phosphatase activity of wild type cells expressing Vph1-mScarlet.

(D) *pho8Δ60 vac8Δ* cells transformed with a plasmid expressing Vph1-mScarlet, Vph1-mScarlet-Atg13M$^{(269-520)}$, overexpressed Vph1-mScarlet-Atg13M$^{(269-520)}$ (OE) or Vph1-mScarlet-Vac8ΔN$^{(19-578)}$ were grown to mid-log phase in SD medium and starved for 1 hour in SD-N medium. Representative fluorescence images are shown.

DIC, differential interference contrast.

Table S1 - Yeast strains used in this study

Name	Genotype	Background	Source
BY4741	his3Δ1 leu2Δ0 met15Δ0 ura3Δ0; Mat a	BY474x	Euroscarf
SEY6210	ura3-52 leu2-3,112 his3-Δ200 trp1-Δ901 lys2-801 suc2-Δ9 mel GAL; Mat alpha	-	Robinson et al., 1988
yAB12	VAC8-4xH3-5xHA:URA; Mat a	BY474x	This study
yAB19	atg13::KAN VAC8-4xH3-5xHA:URA; Mat a	BY474x	This study
yCK36	pep4::NAT atg19::KAN; Mat a	BY474x	This study
yCK286	nvj1::KAN; Mat a	BY474x	This study
yCK566	WT; Mat a	BY474x	Euroscarf
yCK660	atg1::KAN; Mat a	BY474x	Kijanska et al., 2010
yCK801	pho8::pho8Δ60:HIS; Mat a	BY474x	Bas et al., 2018
yDH7	pho8::pho8Δ60:HIS atg1::KAN; Mat a	BY474x	This study
yDH9	pho8::pho8Δ60:HIS vac8::KAN; Mat a	BY474x	This study
yDH34	pho8::pho8Δ60:HIS atg19::KAN vac8::KAN; Mat a	BY474x	This study
yDH42	sfGFP-ATG8 ypt7::NAT atg19::HYG atg1::KAN; Mat a	BY474x	This study
yDH43	sfGFP-ATG8 ypt7::NAT atg19::HYG; Mat a	BY474x	This study
yDH44	sfGFP-ATG8 ypt7::NAT atg19::HYG vac8::KAN; Mat a	BY474x	This study
yDH132	ATG1-GFP:HIS atg8::KAN atg19::HYG; Mat a	BY474x	This study
yDH133	ATG2-GFP:HIS atg8::KAN atg19::HYG; Mat a	BY474x	This study
yDH134	ATG2-GFP:HIS atg8::KAN atg19::HYG vac8::KAN; Mat a	BY474x	This study
yDH135	ATG1-GFP:HIS atg8::KAN atg19::HYG vac8::KAN; Mat a	BY474x	This study
yDH136	ATG1-GFP:HIS atg8::KAN atg19::HYG atg13::KAN; Mat a	BY474x	This study
yDH175	sfGFP-ATG8 atg19::NAT atg1::KAN; Mat a	BY474x	This study
yDH284	VPH1-4xmCherry:URA atg13::KAN atg19::HYG vac8::KAN; Mat a	BY474x	This study
yDH294	ATG2-GFP:HIS atg8::KAN atg19::HYG atg1::KAN; Mat a	BY474x	This study
yDH297	sfGFP-ATG8 atg19::HYG vac8::KAN; Mat a	BY474x	This study
yDH323	VPH1-4xmCherry:URA atg19::HYG vac8::KAN; Mat a	BY474x	This study
yDH325	ATG1-GFP:HIS atg19::HYG vac8::KAN; Mat a	BY474x	This study
yDH327	ATG13-3xsfGFP:URA atg19::HYG vac8::KAN; Mat a	BY474x	This study
yDH333	ape1::KAN atg19::HYG vac8::KAN; Mat a	BY474x	This study
yDH341	ATG1-GFP:HIS atg13::KAN atg19::HYG vac8::KAN; Mat a	BY474x	This study
yDH344	tor1-1 fpr1::URA pho8::pho8Δ60:HIS vac8::KAN Vph1-FRB:NAT; Mat a	BY474x	This study
yDP463	sfGFP-ATG8 atg19::NAT; Mat a	BY474x	Torggler et al., 2016
yFK7	VPH1-4xmCherry:URA atg15::KAN pep4::KAN; Mat alpha	BY474x	This study
yFK10	VPH1-4xmCherry:URA atg15::KAN pep4::KAN vac8::KAN; Mat a	BY474x	This study
yLB61	ATG1-ATG13(567-738)-3xsfGFP:URA atg13::KAN atg19::HYG vac8::KAN; Mat a	BY474x	This study
yLB189	sfGFP-ATG8 pep4::NAT vam3::KAN; Mat a	BY474x	Bas et al., 2018
yML8	pep4::NAT atg19::KAN vac8::KAN; Mat alpha	BY474x	This study
yML13	pep4::NAT atg19::KAN atg1::HIS; Mat alpha	BY474x	This study
yRT247	ATG1-STag:HIS atg8::KAN atg19::HYG; Mat alpha	BY474x	This study
yRT248	ATG1-STag:HIS atg8::KAN atg19::HYG atg13::KAN; Mat alpha	BY474x	This study
yRT249	ATG1-STag:HIS atg8::KAN atg19::HYG vac8::KAN; Mat alpha	BY474x	This study
yRT257	pep4::NAT atg19::KAN atg1::HIS vac8::KAN; Mat alpha	BY474x	This study
yRT261	pho8::pho8Δ60:HIS nvj1::KAN; Mat a	BY474x	This study
yTB434	vac8::KAN; Mat a	BY474x	This study
RGY047	SEC63-GFP:HIS3MX6 ape1::loxP-kanMX-loxP ATG17-yoTagRFP657:KILEU2 atg8::NatMX6; Mat alpha	SEY6210	This study
RGY056	SEC63-GFP:HIS3MX6 ape1::loxP-kanMX-loxP ATG17-yoTagRFP657:KILEU2 atg8::NatMX6 vac8::TRP1; Mat alpha	SEY6210	This study
RGY680	atg8::loxP-kanMX-loxP atg19::TRP1 vac8::hphNT1; Mat alpha	SEY6210	This study

Table S2 - Plasmids used in this study

Name	Characteristics	Promoter	Terminator	Source
pRS315	CEN, LEU2	-	-	Sikorski and Hieter 1989
pRS316	CEN, URA3	-	-	Sikorski and Hieter 1989
pRS406	CEN, URA3	-	-	Sikorski and Hieter 1989
pRS413	CEN, HIS3	-	-	Sikorski and Hieter 1989
pRS415	CEN, LEU2	-	-	Sikorski and Hieter 1989
pRS416	CEN, URA3	-	-	Sikorski and Hieter 1989
pAB19	Pbs2-9xMYC-HKMT; pRS415	PBS2	CYC1	This study
pAC160	Nvj1-9xMYC-HKMT; pRS415	NVJ1	CYC1	This study
pDH1	Vac8(19-578)-GFP [Vac8ΔN-GFP]; pRS415	VAC8	CYC1	This study
pDH2	FKBP-Vac8(19-578)-GFP [FKBP-Vac8ΔN-GFP]; pRS415	VAC8	CYC1	This study
pDH4	mTagBFP2-Ape1; pRS415	APE1	CYC1	This study
pDH8	Atg19(152-191)-Vac8(19-578)-GFP [t-Vac8ΔN-GFP]; pRS415	VAC8	CYC1	This study
pDH9	Atg19(152-191)-Vac8(19-578)-mTagBFP2 [t-Vac8ΔN-BFP]; pRS415	VAC8	CYC1	This study
pDH10	Atg19(152-191)-Vac8(19-578)-GFP [t-Vac8ΔN-GFP]; pRS413	VAC8	CYC1	This study
pDH11	Vac8(19-578)-GFP [Vac8ΔN-GFP]; pRS413	VAC8	CYC1	This study
pDH12	Vac8-GFP; pRS413	VAC8	CYC1	This study
pDH13	Vac8(19-578)-mTagBFP2 [Vac8ΔN-BFP]; pRS415	VAC8	CYC1	This study
pDH14	Atg19(152-191)-Atg13(567-738)-mTagBFP2 [t-Atg13C-BFP]; pRS415	VAC8	CYC1	This study
pDH32	Vph1-mScarlet-Atg13(269-520) [Vph1-mScarlet-Atg13M (OE)]; pRS416	VAC8	CYC1	This study
pDH33	Vph1-mScarlet; pRS416	VAC8	CYC1	This study
pDH39	Vph1-mScarlet-Vac8(19-578) [Vph1-mScarlet-Vac8ΔN]; pRS416	VAC8	CYC1	This study
pDH41	Vph1-mScarlet-Atg13(269-520) [Vph1-mScarlet-Atg13M]; pRS416	ATG13	CYC1	This study
pMS5	Vac8-GFP; pRS415	VAC8	CYC1	This study
pMS13	Vac8-C4G-C5T-C7S-GFP [Vac8Δpal-GFP]; pRS415	VAC8	CYC1	This study
pMS130	Vac8-G2A-GFP [Vac8Δmyr-GFP]; pRS415	VAC8	CYC1	This study
pMS131	Vac8-G2A-C4G-C5T-C7S-GFP [Vac8Δac-GFP]; pRS415	VAC8	CYC1	This study
pRT118	Atg13-9xMYC-HKMT; pRS415	ATG13	CYC1	This study
pRT119	Atg13(1-520)-9xMYC-HKMT [Atg13ΔC-HKMT]; pRS415	ATG13	CYC1	This study
-	mCherry-V5-Atg8; pRS406	CUP1	CYC1	Mari et al., 2010
-	Pgk1-GFP; pRS316	PGK1	ADH1	Welter et al., 2010

Journal of Cell Science • Supplementary information

Table S3 - List of yeast strains and plasmids used per figure

Figure	Yeast strain	Plasmids
Figure 1A	BY4741	-
Figure 1A	yCK660	-
Figure 1A	yTB434	-
Figure 1B	yCK801	-
Figure 1B	yDH7	-
Figure 1B	yDH9	-
Figure 1C	BY4741	-
Figure 1C	yCK660	-
Figure 1C	yTB434	-
Figure 1E and 1F	yDH132	-
Figure 1E and 1F	yDH136	-
Figure 1E and 1F	yDH135	-
Figure 1G	yRT247	-
Figure 1G	yRT248	-
Figure 1G	yRT249	-
Figure 1H	yCK801	-
Figure 1H	yDH7	-
Figure 1H	yRT261	-
Figure 1J	yAB19	pAB19
Figure 1J	yAB19	pRT118
Figure 1J	yAB19	pAC160
Figure 1J	yAB12	pAC160
Figure 1J	BY4741	pRT118
Figure 1J	BY4741	pAC160
Figure 2A and 2B	yDH133	-
Figure 2A and 2B	yDH294	-
Figure 2A and 2B	yDH134	-
Figure 2C and 2D	yDH43	-
Figure 2C and 2D	yDH42	-
Figure 2C and 2D	yDH44	-
Figure 2F	yDP463	-
Figure 2F	yDH175	-
Figure 2F	yDH297	-
Figure 3A, 3B, 3C and 3D	yCK36	-
Figure 3A, 3B, 3C and 3D	yML8	-
Figure 3A, 3B, 3C and 3D	yML13	-
Figure 3A, 3B, 3C and 3D	yRT257	-
Figure 3E and 3F	yLB189	-
Figure 3E and 3F	yFK7	-
Figure 3E and 3F	yFK10	-

Figure	Yeast strain	Plasmids
Figure 4A	RGY680	mCherry-V5-ATG8, pMS5
Figure 4A	RGY680	mCherry-V5-ATG8, pMS13
Figure 4A	RGY680	mCherry-V5-ATG8, pMS130
Figure 4A	RGY680	mCherry-V5-ATG8, pMS131
Figure 4B	yCK801	pRS415
Figure 4B	yDH7	pRS415
Figure 4B	yDH9	pRS415
Figure 4B	yDH9	pMS5
Figure 4B	yDH9	pMS13
Figure 4B	yDH9	pMS130
Figure 4B	yDH9	pMS131
Figure 4C, 4D and 4E	RGY047	mCherry-V5-ATG8
Figure 4C, 4D and 4E	RGY056	mCherry-V5-ATG8
Figure 5A	yDH323	pDH1
Figure 5A	yDH323	pMS5
Figure 5C	yDH323	pDH4, pDH10
Figure 5C	yDH323	pDH4, pDH11
Figure 5C	yDH333	pRS415, pDH10
Figure 5D and 5E	yDH327	pDH9
Figure 5D and 5E	yDH327	pDH13
Figure 6A	BY4741	pRT118
Figure 6A	yAB19	pAB19
Figure 6A	yAB19	pRT118
Figure 6A	yAB19	pRT119
Figure 6B and 6C	yDH325	pDH9
Figure 6B and 6C	yDH341	pDH9
Figure 6B and 6C	yLB61	pDH9
Figure 7A, 7B, S4A and S4B	yDH284	pDH14, pDH11
Figure 7A, 7B, S4A and S4B	yDH284	pDH14, pRS413
Figure 7A, 7B, S4A and S4B	yDH284	pDH14, pDH12
Figure 7C	yDH34	pMS5
Figure 7C	yDH34	pDH1
Figure 7C	yDH34	pDH8
Figure 7E	yDH344	pMS5
Figure 7E	yDH344	pDH2
Figure 7F	yDH344	pMS5
Figure 7F	yDH344	pRS415
Figure 7F	yDH344	pDH2

Journal of Cell Science • Supplementary information

Figure	Yeast strain	Plasmids
Figure S1	yCK566	Pgk1-GFP
Figure S1	yCK660	Pgk1-GFP
Figure S1	yCK286	Pgk1-GFP
Figure S1	yTB434	Pgk1-GFP
Figure S2	yLB189	-
Figure S2	yFK7	-
Figure S2	yFK10	-
Figure S3A	BY4741	pRS415
Figure S3A	yCK660	pRS415
Figure S3A	yTB434	pRS415
Figure S3A	yTB434	pMS5
Figure S3A	yTB434	pMS13
Figure S3A	yTB434	pMS130
Figure S3A	yTB434	pMS131
Figure S3B and S3C	RGY680	mCherry-V5-ATG8, pMS5
Figure S3B and S3C	RGY680	mCherry-V5-ATG8, pRS415
Figure S3B and S3C	RGY680	mCherry-V5-ATG8, pMS131
Figure S4C	yCK801	pDH33
Figure S4C	yCK801	pRS316
Figure S4C	yCK801	pDH41
Figure S4C	yCK801	pDH32
Figure S4C	yCK801	pDH39
Figure S4C and S4D	yDH9	pDH33
Figure S4C	yDH9	pRS316
Figure S4C and S4D	yDH9	pDH41
Figure S4C and S4D	yDH9	pDH32
Figure S4C and S4D	yDH9	pDH39
